# Effect of GARP on osteogenic differentiation of bone marrow mesenchymal stem cells via the regulation of TGFβ1 *in vitro*

**DOI:** 10.7717/peerj.6993

**Published:** 2019-05-23

**Authors:** Ruixue Li, Jian Sun, Fei Yang, Yang Sun, Xingwen Wu, Qianrong Zhou, Youcheng Yu, Wei Bi

**Affiliations:** Department of Stomatology, Fudan University Zhongshan Hospital, Shanghai, Shanghai, China

**Keywords:** GARP, TGFβ1, Bone marrow mesenchymal stem cell, Bone regeneration

## Abstract

Mesenchymal stem cells (MSCs), which have multipotential differentiation and self-renewal potential, are possible cells for tissue engineering. Transforming growth factor β1 (TGFβ1) can be produced by MSCs in an inactive form, and the activation of TGFβ1 functions as an important regulator of osteogenic differentiation in MSCs. Recently, studies showed that Glycoprotein A repetitions predominant (GARP) participated in the activation of latent TGFβ1, but the interaction between GARP and TGFβ1 is still undefined. In our study, we successfully isolated the MSCs from bone marrow of rats, and showed that GARP was detected in bone mesenchymal stem cells (BMSCs). During the osteogenic differentiation of BMSCs, GARP expression was increased over time. To elucidate the interaction between GARP and TGFβ1, we downregulated GARP expression in BMSCs to examine the level of active TGFβ1. We then verified that the downregulation of GARP decreased the secretion of active TGFβ1. Furthermore, osteogenic differentiation experiments, alkaline phosphatase (ALP) activity analyses and Alizarin Red S staining experiments were performed to evaluate the osteogenic capacity. After the downregulation of GARP, ALP activity and Alizarin Red S staining significantly declined and the osteogenic indicators, ALP, Runx2, and OPN, also decreased, both at the mRNA and protein levels. These results demonstrated that downregulated GARP expression resulted in the reduction of TGFβ1 and the attenuation of osteoblast differentiation of BMSCs *in vitro*.

## Introduction

Implant supported dentures have become a mainstream treatment in repairing dentition defects and deletions (Alkan et al., 2018; Greenberg, 2017). However, anodontia caused by serious decay, periodontal disease, congenital anodontia, and trauma may result in severe alveolar bone resorption, which can be a disadvantage of implantation. Although autogenous bone grafts are generally thought to be the gold standard for bone regeneration (Moses et al., 2007; Sbordone et al., 2014; Zhang et al., 2014), this procedure is limited due to local complications and an insufficiency of bone volume in donor sites (Jensen, Jensen & Worsaae, 2016; Nkenke & Neukam, 2014). A novel and easily available treatment is therefore necessary for bone regeneration. Mesenchymal stem cells (MSCs), which can be isolated from tissues such as blood, adipose tissue, bones, and teeth, have the multipotential to differentiate into osteoblasts, chondrocytes, and adipocytes, and have become a promising vector for bone tissue engineering (Ma et al., 2017). Because they are easy to cultivate and expand, and maintain their pluripotency after serial subcultivation *in vitro*, bone marrow MSCs (BMSCs) are an ideal vehicle for tissue regeneration in severe bone defects and bone remodeling (Yang, FMV & Putnins, 2010; Han et al., 2013).

However, bone formation is a complex process controlled by many factors (Loeffler et al., 2017; Schroeder & Mosheiff, 2011), especially transforming growth factor β1 (TGFβ1). TGFβ1, which is secreted by bone marrow stromal cells and hematopoietic progenitors, is abundant in bone matrix, and functions as a modulator of cell growth, inflammation, and matrix synthesis (Kirk & Kahn, 1995; Kim & Niyibizi, 2001; Rahman et al., 2015; Majidinia, Sadeghpour & Yousefi, 2018), so we assumed that the use of TGFβ1 may be an alternative approach in stem cell-based tissue engineering. However, TGFβ1 is secreted as an inactive complex, which includes the mature TGFβ1 dimer and the latency-associated proteins (LAPs). Furthermore, LAPs remain inactive. It is the removal of TGFβ1 from LAPs that activates the TGFβ1 function. It is generally accepted that the latent TGFβ1-binding proteins (LTBPs) participate in the transportation and activation process. LTBPs, associated with latent TGFβ1, assist the complex transfer and then are anchored to the extracellular matrix (ECM). The involvement of protease, thrombospondin-1, and integrin, and changes in the condition of reactive oxygen species and pH then trigger the activation process of latent TGFβ1.

Another hypothesis is that membrane Glycoprotein A repetitions predominant (GARP) also participates in the activation of TGFβ1. GARP, which encodes a leucine-rich repeat containing 32 (LRRC32) protein, was first isolated from a breast carcinoma (Ollendorff et al., 1992). Most proteins of GARP with the LRR motif are membrane bound, and the remaining proteins can be secreted to extracellular sites or are localized to the cytoplasm or nucleus (Rothberg et al., 1990; Ohkura & Yanagida, 1991). Many relevant studies have focused on the biological function of GARP, and have proposed that GARP is involved in the activation of latent TGFβ1. It was reported that GARP and latent TGFβ1 are co-localized on the membrane of BMSCs (Carrillo-Galvez et al., 2015; Niu et al., 2017), but the interaction between them and whether GARP regulates the bioactivities of TGFβ1 in BMSCs still remain to be elucidated. Furthermore, whether GARP can affect the osteogenic differentiation ability of BMSCs is unknown. We therefore downregulated the expression of GARP in BMSCs, and found that the secretion of TGFβ1 was decreased, and the osteogenic results showed attenuated osteogenesis while the expression of GARP was knocked-down.

## Materials and Methods

### Animals

All the BMSCs were isolated from 4-week-old male Sprague-Dawley (SD) rats under SPF raising conditions. The experiments were approved by the Animal Research Committee of Zhongshan Hospital, Fudan University, Shanghai, China (2016-128).

### Isolation and culture of BMSCs

Rats were sacrificed by cervical dislocation. The femur and tibia were isolated, and both ends of the bones were removed to expose the marrow, which was flushed with a 10 mL syringe filled with alpha-Minimum Essential Medium (α-MEM; Gibco, Thermo Fisher Scientific, Waltham, MA, USA), 10% fetal bovine serum (FBS; Gibco; Thermo Fisher Scientific), and 1% penicillin/streptomycin. The fresh marrow suspension was filtered through a 70 µm cell strainer and centrifuged at 500 × g for 5 min. The supernatant was discarded, and the sediment was resuspended and seeded in 25 cm^2^ culture flasks. The culture medium was replaced after 48 h. BMSCs were trypsinized using 0.25% trypsin/1mM EDTA (Gibco; Thermo Fisher Scientific) to subculture the cells when they grew to 80% confluency. Cells at passages 3–6 were used for all experiments.

### Identification and GARP expression of rat BMSCs by using flow cytometry

To identify the target cell, we used fluorescein isothiocyanate (FITC)-conjugated CD90 (561973; 1:100; BD biosciences; Franklin, Lakes, NJ, USA), CD45(561867; 1:100; BD biosciences), CD105(NB500-453; 1:100; novus biologicals; USA) and phycoerythrin (PE)-conjugated CD 44 (MA5-16908; 1:100; Thermo Fisher Scientific Inc.) to label the BMSC membranes. For analysis of GARP expression, we used a LRRC32/GARP antibody (NBP2-24664; 1:1,000; Novus Biologicals) followed by Alexa Fluor 647 AffiniPure secondary antibody (111-605-003; 1:100; Jackson Immuno Research Laboratories, West Grove, PA, USA). When cells grew to 80% confluency, they were harvested by gently washing once with phosphate-buffered saline (PBS), trypsinizing for 2 min at 37 °C , and centrifuging for 5 min at 500 × g. The cells were resuspended to a concentration of 1 ×10^6^ cells/mL in PBS, and 100 µL suspensions were used for each sample. One µg of antibody was added to each sample, then incubated on ice in the dark for 1 h. One mL of PBS was added to stop the reaction, then the suspension was centrifuged for 5 min at 500 × g, and the supernatant was discarded. The pellet was washed twice by resuspending the cells in PBS and centrifuged for 5 min at 500 × g, followed by discarding the supernatant. The pellet was resuspended in 500 µL of PBS per sample, and used for the flow cytometry (BD Biosciences) analysis.

### Immunofluorescent staining

The third passage BMSCs were seeded in a 6-well culture plate with prepared cell sheets at a density of 1. 5 ×10^4^ cells/mL, and the cells were incubated overnight. When the cells were 50% confluent, the cell sheets were washed three times by PBS. The cells were then fixed with 4% formalin for 30 min, followed by washing three times with PBS, and blocked with 5% bovine serum albumin for 30 min. The experimental and control group cells were then incubated with anti-GARP antibody (NBP2-68740; 1:100; Novus Biologicals) and homologous anti-IgG antibody (A7016; 1:100; Beyotime Institute of Biotechnology, Haimen, China) at 4 °C in the dark overnight respectively, and the cell sheets washed three times with PBS. The cells were then incubated with FITC secondary antibody (A0562; 1:100; Beyotime Institute of Biotechnology) at room temperature for an hour. Cell sheets were washed three times with PBS. Then cells were incubated with 4′, 6-diamidine-2′-pheynylindole dihydrochloride (DAPI; Invitrogen, Carlsbad, CA, USA) for 10 min in the dark, and then washed as previously described. The cell sheets were imaged using an immunofluorescence microscope (Olympus, Tokyo, Japan).

### Lentivirus gene vector production and transfection of BMSCs

The GARP short hairpin RNA (shRNA)-encoded lentivirus vector was packaged by Shanghai Hanyin (Shanghai, China). Lentiviral vectors for rat GARP-shRNA carried a green fluorescent protein (GFP) sequence. The target sequences for rat GARP knockdown are listed in Table 1. The recombinant GARP-shRNA lentivirus and the negative control lentivirus were prepared and titered to 10^9^ TU/mL (transfection unit). Before transfection, BMSCs were seeded at a density of 5 × 10^4^ cells per well in a 6-well culture plate and incubated overnight. The cells were approximately 50% confluent on the day of infection. A mixture of medium with polybrene (Shanghai Hanyin) was prepared at a final concentration of 5 µg/mL. The medium was removed from the plate wells and replaced with 1 mL of this polybrene/media mixture. The cells were infected by adding 10 µL shRNA lentivirus to the culture. The plate was gently swirled to mix and incubated in 5% CO_2_ at 37 °C. The medium was changed after 8 h. Stable clones expressing a GARP shRNA (GARP-sh) were selected using 3 µg/mL puromycin dihydrochloride (Shanghai Hanyi). At the same time, negative control cells (NC) were infected by the same dose of empty vector virus.

**Table 1:** Primers for RT-qPCR and GARP shRNA sequences. This table showed the primers for RT-qPCR in the present study and GARP shRNA sequences.

Gene	Forward (3′–5′)	Reverse (5′–3′)
GARP	GGCAGAGAACAGCCTCACTC	AAGGCACCATCCTCAATGTC
ALP	GATGGACAAGTTCCCCTTTG	CCTTCACGCCACACAAGTAG
Runx2	CCTCTGACTTCTGCCTCTGG	CCTCTGACTTCTGCCTCTGG
OPN	CCAAGCGTGGAAACACACAGCC	GGCTTTGGAACTCGCCTGACTG
β-actin	GCAGGAGTACGATGAGTCCG	ACGCAGCTCAGTAACAGTCC
GARP-sh1		GGCTCAACCTACAGGGAAA
GARP-sh2		GGTTAAAGGCTCAGAGAAC
GARP-sh3		GGCTGTACTTGCAGGGAAA
GARP-sh4		GCACTTCGCCACCTGGATTTA
GARP-sh5		GCAACAGCATTGAGACCTTCC

**Notes.**

GARP Glycoprotein A repetitions predominant ALP Alkaline phosphatase OPN Osteopontin Runx2 Runt-related transcription factor 2

### Cell Counting Kit-8 (CCK-8) analysis of transfected BMSCs

To further investigate the differences of proliferation and viability between transfected and controlled BMSCs, CCK-8 experiments were performed. The transfected and controlled BMSCs were seeded in a 96-well culture plate at a density of 1 × 10^3^ cells/mL. At the same time, there were six wells with only culture medium, which were used as the blank controls. The plates were then incubated at 37 °C. According to the manufacturer’s instructions, 10 µL CCK-8 solution (Dojindo Molecular Technologies, Kumamoto, Japan) was added to each well. The optical density (OD) value was measured at a wavelength of 450 nm, with the results presented as the mean.

### Quantitative real time-polymerase chain reaction (RT-qPCR)

Extractions of total RNA were obtained from each group using TRIzol (Invitrogen, Thermo Fisher Scientific, Waltham, MA, USA) according to manufacturer’s instructions. Reverse transcription of RNA was conducted using the PrimeScript RT Master Mix (Takara Bio, Ostu, Japan). RT-qPCR analyses were performed by using the SYBR-Green Real-Time PCR Master Mix (Takara Bio). Rat β-actin, a single housekeeping gene, was selected as an internal control for normalization. All primers used in the RT- qPCR are listed in Table 1. The RT-qPCR procedure was conducted using the following program: one cycle at 95 °C for 30 s; 40 cycles at 95 °C for 5 s; and one cycle at 60 °C for 30 s. The total volume of the reaction was 20 µL, and the relative expression of the target gene was analyzed by the comparative cycle threshold (2^−^^ΔΔ*Ct*^) method.

### Western blot analysis

For western blot analysis, cells from each group were washed with PBS on ice. Cells were lysed using RIPA lysis buffer (Beyotime Institute of Biotechnology). The lysate was collected and then centrifuged at 12,000× g for 5 min. The supernatant was collected and the protein concentration was determined using a BCA protein assay kit (Beyotime Institute of Biotechnology). Twenty µg of protein lysate from each sample was resolved using a 10% SDS-PAGE gel and then transferred to a polyvinylidene fluoride membrane. The membranes were blocked with 5% nonfat milk for 2 h at room temperature (RT), and the membrane was incubated with primary antibodies including anti-GARP (NBP2-24664; 1:1,000; Novus Biologicals), anti-ALP (08337; 1:10,000; Abcam, Cambridge, UK), anti-OPN (91655; 1:1,000; Abcam), anti-Runx2 (23981; 1:1,000; Abcam), and anti- β-actin (8227; 1:1,000; Abcam) at 4 °C overnight. The next day, the membranes were washed in TBST (20 mM Tris-HCL, 137 mM NaCl and 0.1% Tween-20; pH 7.6) three times, for 10 min each time. The membrane was then blocked with goat-anti-rabbit IgG-HRP antibody (SC-2370; 1:5,000; Santa Cruz Biotechnology, Dallas, TX, USA) for 1 h, followed by washing in TBST three times, with 10 min for each wash. Proteins were detected by Chemiscope5600 (Shanghai Clinx Science Instruments, Shanghai, China) using BeyoECL Plus Reagent (Beyotime Institute of Biotechnology).

### TGFβ1 analysis by ELISA of transfected BMSCs

When cells grew to 80% confluency, the medium was changed to SD rat mesenchymal stem cell osteogenic differentiation medium (Cyagen Biosciences, Santa Clara, CA, USA). Supernatants were collected at 6 h, 12 h, 18 h, 24 h, and 48 h after the change of medium. All samples were centrifuged at 2,500 rpm/min for 20 min, and cell debris was discarded to obtain the supernatant. TGFβ1 levels were analyzed using a rat TGFβ1 ELISA kit (Dakewe Bioengineering, Shenzhen, China), according to the manufacturer’s instructions.

### Osteogenic differentiation

Cells were seeded at a density of 5 × 10^4^ cells per well in a 6-well culture plate and incubated. When cells grew to 80% confluency, the medium was replaced with SD rat mesenchymal stem osteogenic differentiation medium (Cyagen Biosciences). The differentiation medium was changed every 3 days.

### Alkaline phosphatase (ALP) activity analysis

After osteogenic differentiation, cells were washed gently with PBS, fixed with 4% formalin for 30 min, and stained with a BCIP/NBT alkaline phosphatase color development kit (Beyotime Institute of Biotechnology) for 30 min at RT. Finally, they were washed with PBS and then visualized with a microscope (Olympus, Tokyo, Japan).

### Alizarin Red S staining experiment

On the days 14 after the osteogenic differentiation, cells were gently washed with PBS, fixed with 4% formalin for 30 min and then washed with ddH_2_O. Each well was added about 1mL Alizarin Red S staining reagent (Cyagen Biosciences) for 3–5 min at RT. Then cells were gently washed with ddH_2_O and visualized with a microscope (Olympus, Tokyo, Japan).

### Statistical analysis

All data are expressed as the mean ± standard deviation. *P* values < 0.05 were considered as statistically significant differences. The comparisons of two groups were performed using the two-tailed *t*-test, and the results of more than two groups were analyzed by one-way analysis of variance. Statistical analysis was performed using Prism 6.0 software (GraphPad, La Jolla, CA, USA).

## Results

### Identification of rat BMSCs and GARP expression of BMSCs

**Figure 1:**
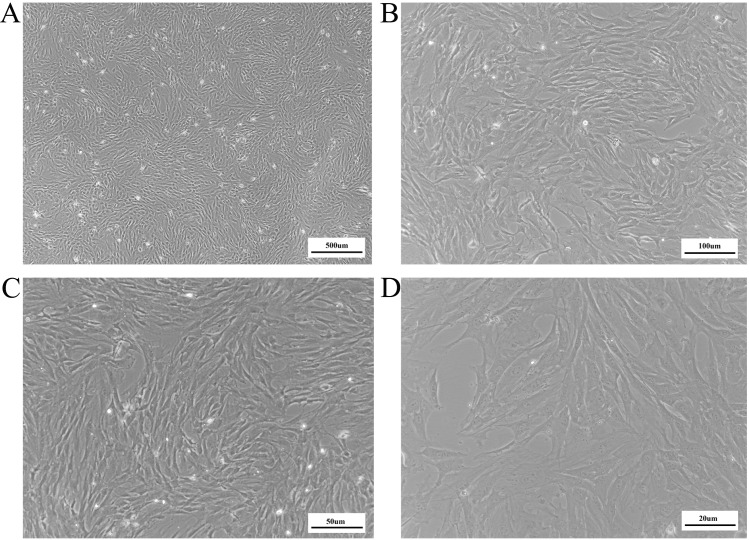
BMSCs morphological characters observed under light microscope. The images revealed that BMSCs showed a spindal shape and typical fibrablast appearance. BMSCs, bone marrow mesenchymal stem cells. (A) 40X. Bar = 500 µm (B) 100X. Bar = 100 µm (C) 200X. Bar = 50 µm (D) 400X. Bar = 20 µm.

**Figure 2:**
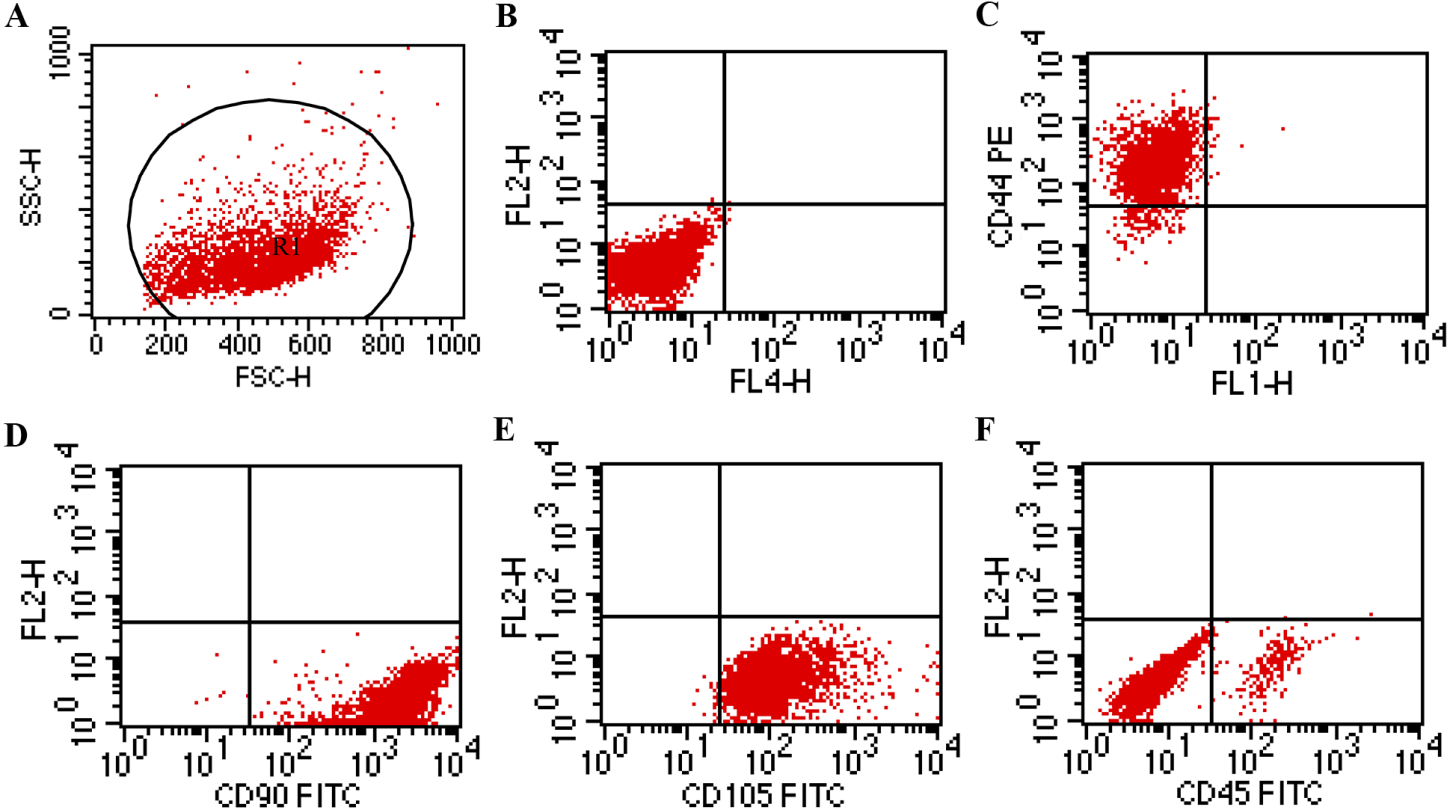
Cytometry results of BMSCs. Cytometry results demonstrated that BMSCs were positive for CD 44 (95.82%), CD 90 (99.85%), CD 105 (99.09%) and negative for CD 45 (5.53%). BMSCs, bone marrow mesenchymal stem cells; FITC, fluorescein isothiocyanate; PE, phycoerythrin. (A) count; (B) Blank Control; (C) CD 44; (D) CD 90; (E) CD 105; (F) CD 45; (*t* test).

**Figure 3:**
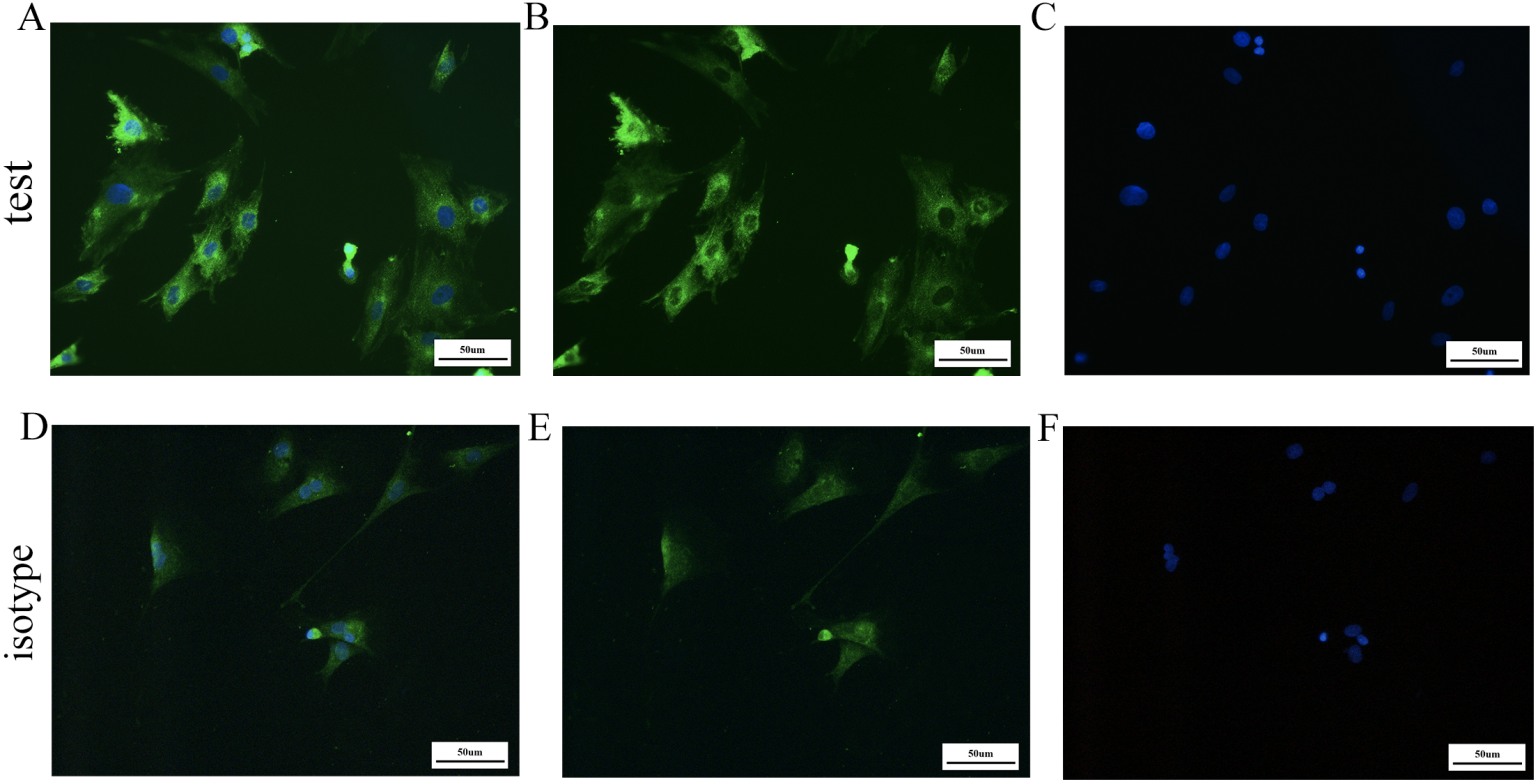
Immunofluorescent staining experiment. For immunofluorescent staining experiment, we used GARP antibody to show the expression of GARP protein, homologous anti-IgG antibody as isotype control and DAPI to show the location of nucleus. GARP, Glycoprotein A repetitions predominant; DAPI, diamidino-phenyl-indole. (A) and (D) showed the merge image of two groups; (B) and (E) showed the FITC immunofluorescent staining; (C) and (F) showed the DAPI staining; 200X; Bar = 50 µm.

The mature BMSCs adhered to the bottom of the culture flask and showed a spindle shape and typical fibroblast-like appearance (Fig. 1). To identify the purity of BMSCs, we assessed the expression of bone marrow mesenchymal and hematopoietic stem cell membrane epitopes, CD44, CD90, CD105 and CD45. Based on the cytometry results (Fig. 2), positive percentage of BMSCs for the CD44 was 95.82%, the CD90 99.85%, the CD105 99.09%, while the CD45 positive ratio was 5.53%. This indicated that we obtained a high purity of BMSCs. For the detection of GARP expression of BMSCs, we used anti-GARP antibody to react with GARP protein, homologous anti-IgG antibody as isotype control, and FITC-conjugated secondary antibody for immunofluorescence staining, followed by imaging with a fluorescence microscope, which showed that GARP was localized to both the membrane and cytoplasm of BMSCs (Fig. 3).

**Figure 4:**
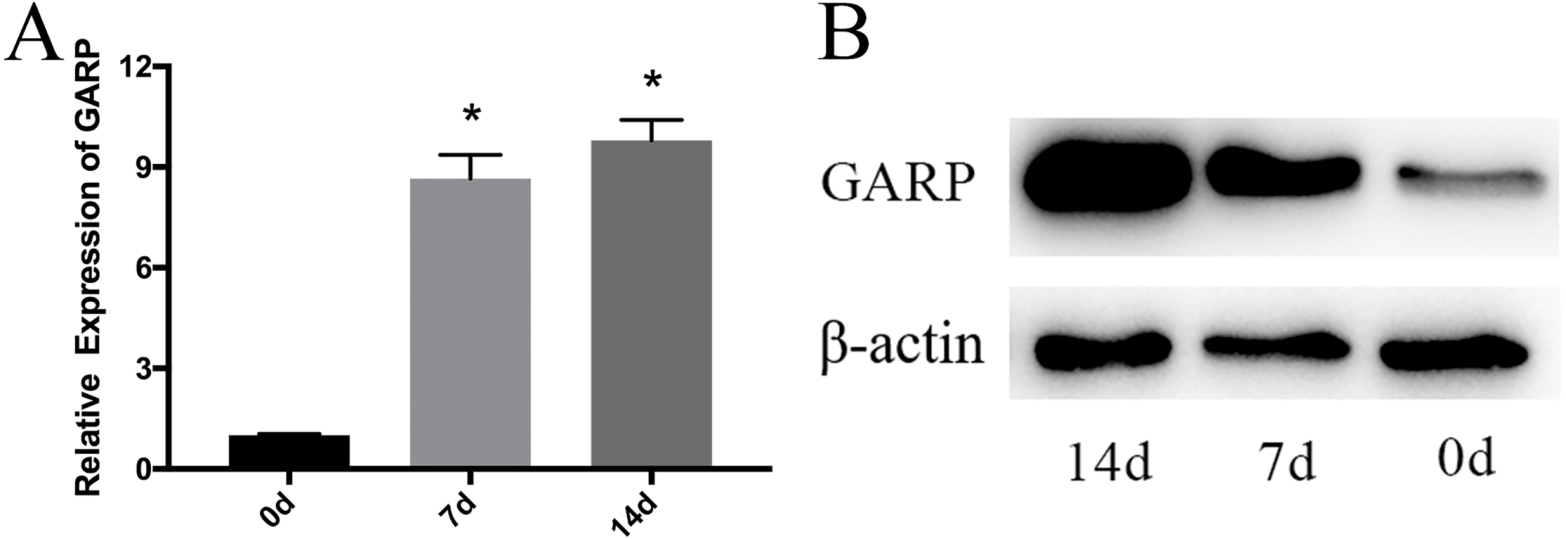
GARP expression during osteogenic differentiation. According to the qRT-PCR and western blot experiments results, on days 7 and 14 after osteogenic differentiation, GARP mRNA and protein levels increased significantly, compared to the days 0. (A) qRT-PCR experiment of GARP mRNA expression; (B) western blot experiment of GARP protein expression; (*t* test, * *P* < 0.05).

To verify the expression level of GARP during osteogenic differentiation, RT-qPCR and western blot experiments were performed at different stages of osteogenic differentiation on days 0, 7 and 14. GARP expression levels increased significantly with the increasing osteogenic differentiation time, indicating that GARP may play an important role in osteogenic differentiation (Fig. 4).

### GARP expression was knocked-down by lentivirus transfection

**Figure 5:**
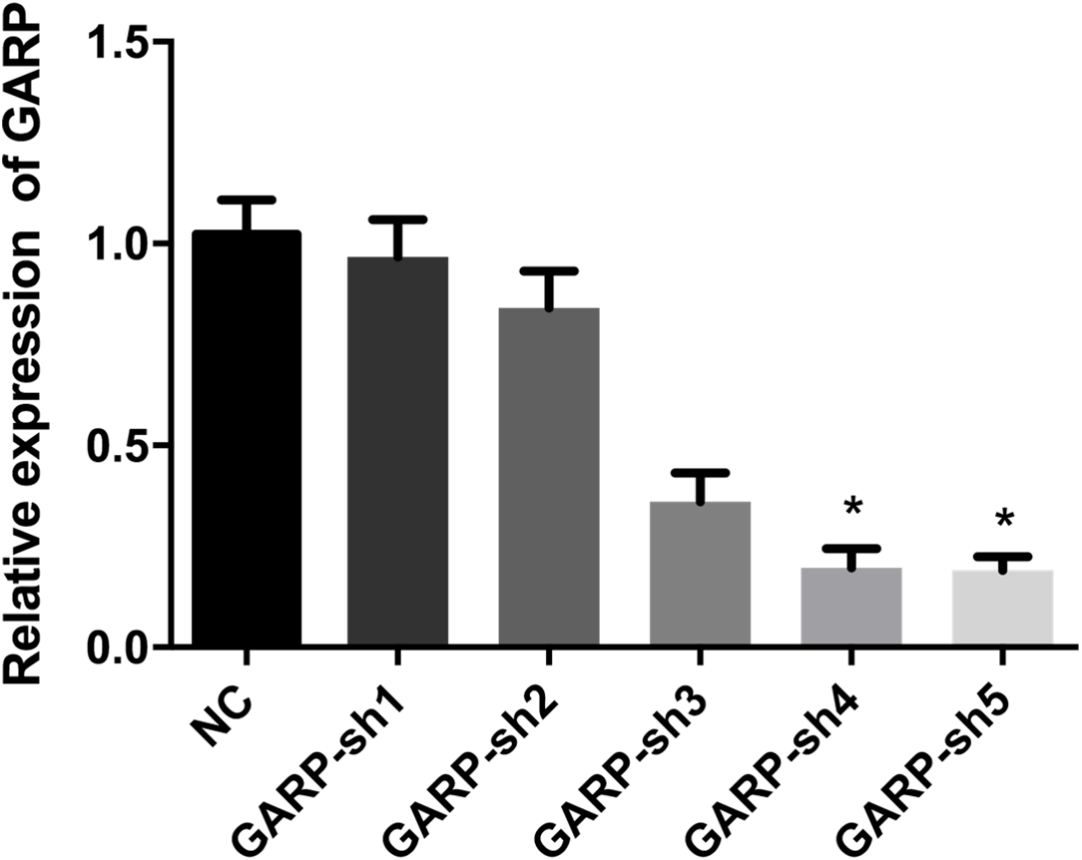
qRT-PCR results of GARP expression. qRT-PCR results showed that GARP-sh4 and GARP-sh5 shRNA could significantly down-regulate the mRNA expression of GARP in BMSCs.(*t* test, * *P* < 0.05).

**Figure 6:**
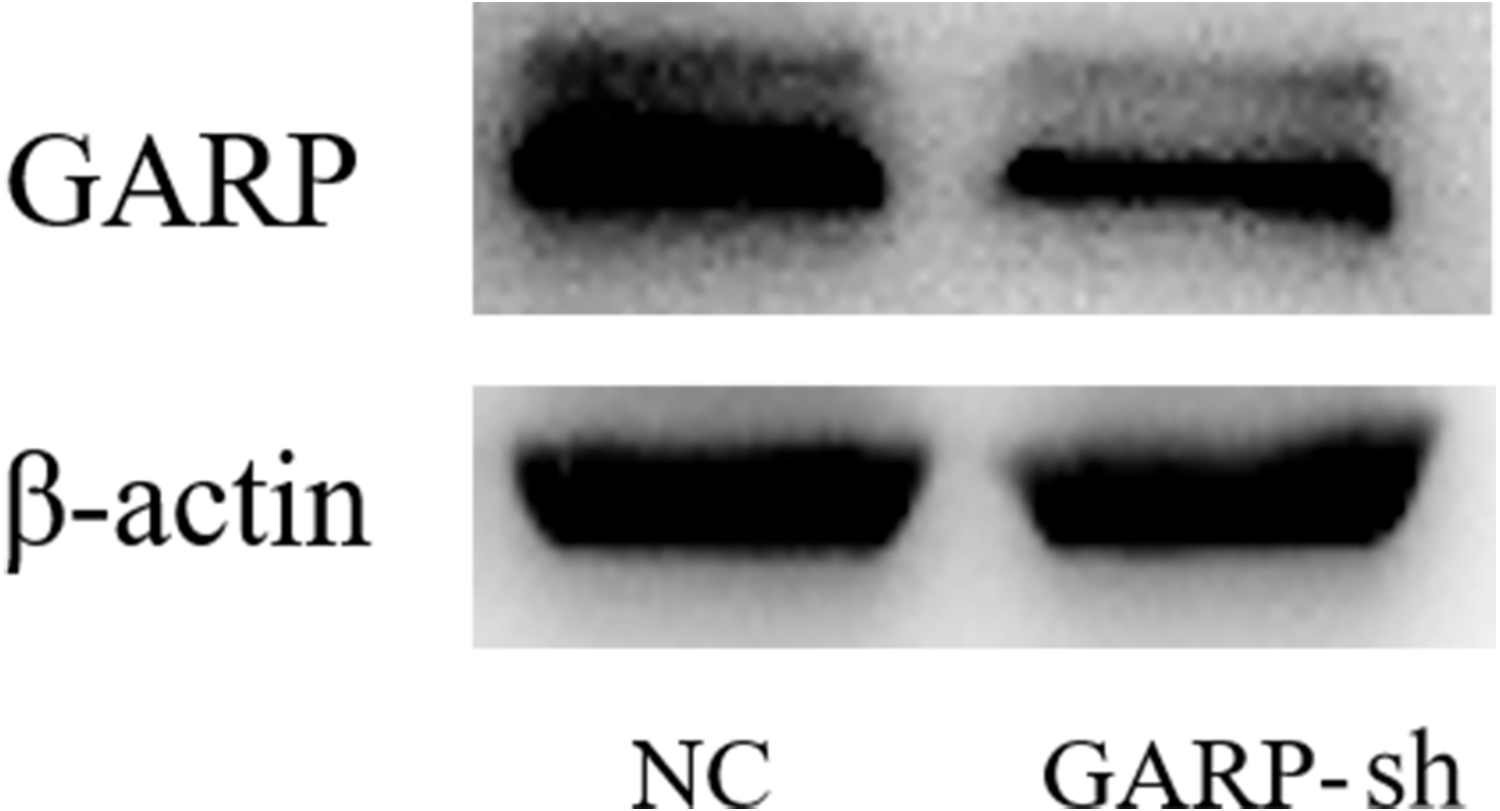
Western blot experiments of GARP expression. Western blot experiments showed that GARP total protein expression in GARP-sh group was decreased, compared to control group.

**Figure 7:**
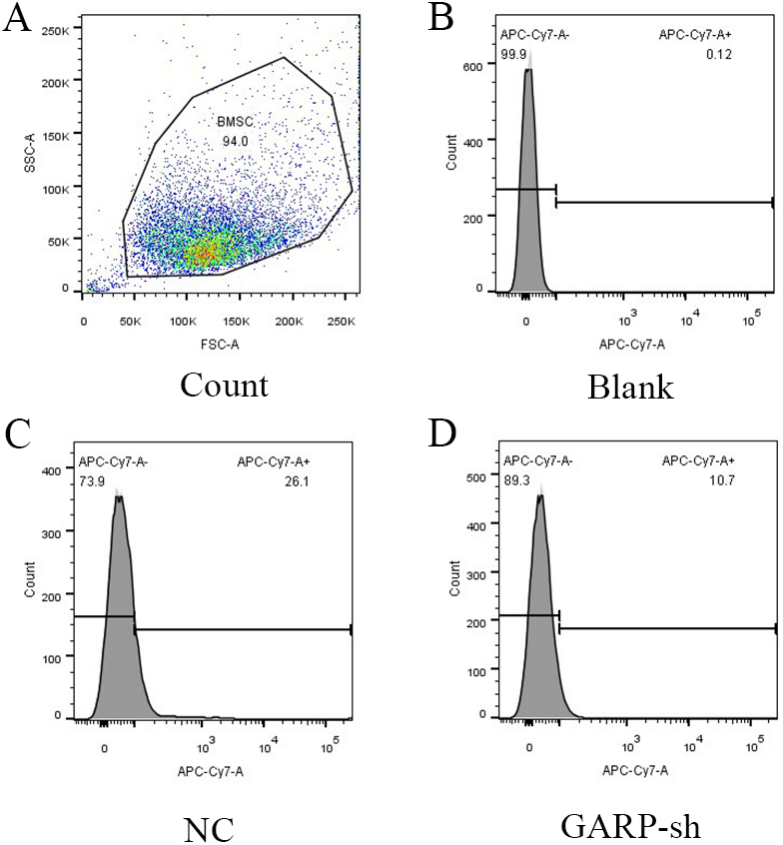
Cytometry results of GARP expression. Cytometry results demonstrated that the GARP expression was 26.1% in NC group and 10.7% in GARP-sh group. (*t* test); (A) the count number of tested cells; (B) blank group; (C) NC group; (D) GARP-sh group.

To further investigate the bioactivities of GARP, we constructed the GARP-shRNA encoded lentivirus vector to silence the expression of GARP. After selection of successfully transfected cells by puromycin dihydrochloride, samples were extracted to analyze the GARP mRNA and protein levels via RT-qPCR and western blotting, respectively. We tested five targets for RNA interference to choose the most the effective one. Based on the RT-qPCR results, mRNA levels of GARP were significantly decreased with transfection of GARP-sh4 and GARP-sh5 (Fig. 5). The subsequent experiments were therefore conducted using GARP-sh5. GARP total protein expression levels were examined by western blotting. The results showed a dramatic decline of total protein expression (Fig. 6). We hypothesized that the membrane localized GARP, which bound latent TGFβ1 to the cell surface, was a key component in regulating the bioactivities of BMSCs, so we performed flow cytometry to determine GARP membrane protein levels of the treatment and control groups, which showed that the GARP membrane protein expression level in the NC group was 26.1%, and 10.7% in the GARP-sh group. There was a 15.4% expression difference between the transfected and NC groups (Fig. 7).

**Figure 8:**
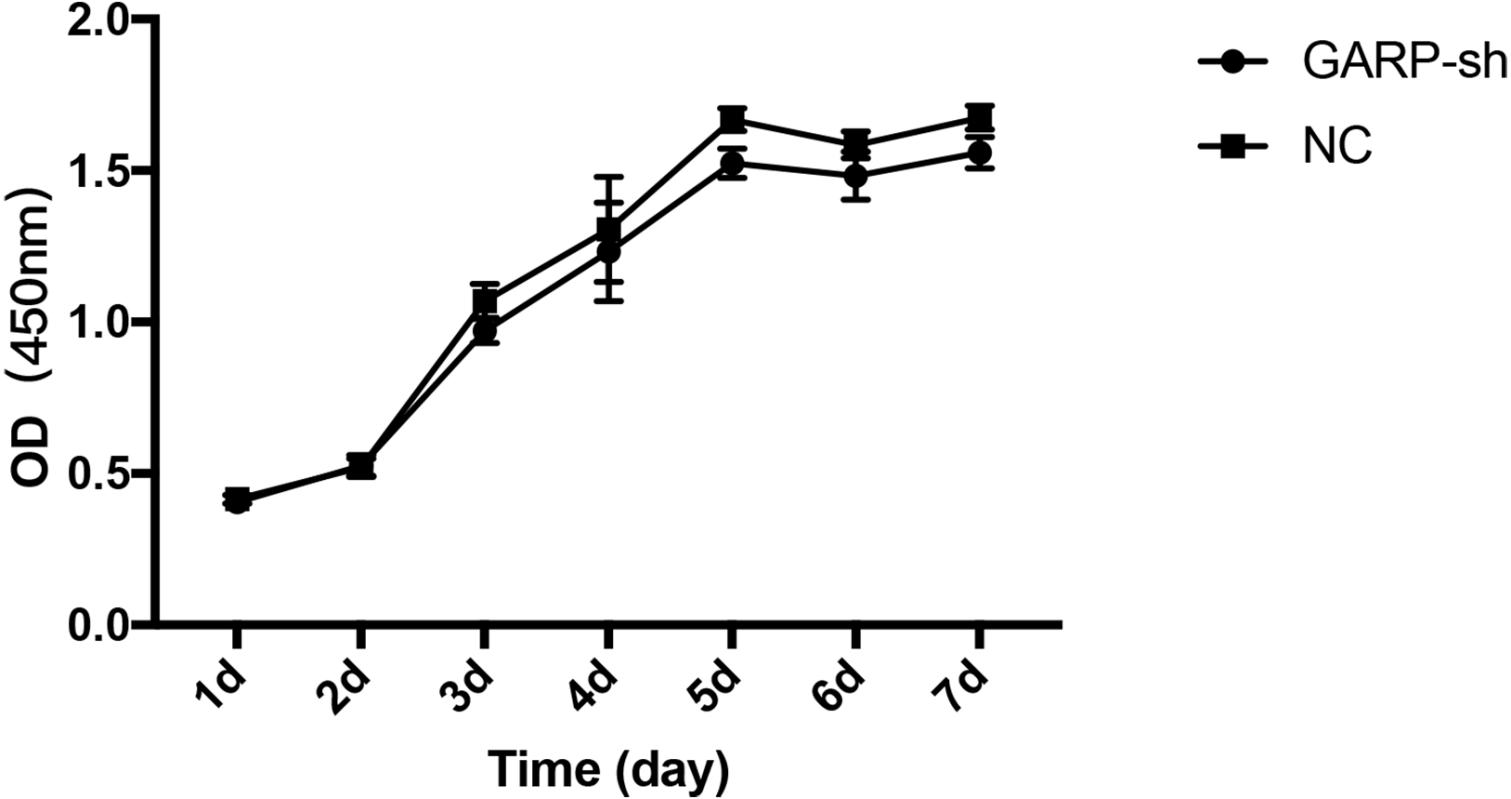
Cell Counting Kit-8 (CCK-8) analysis of transfected BMSCs. CCK-8 analysis result demonstrated that cells of GARP-sh group and NC group were in the slow growth period on the first two days after seeding in the plate. On the days 3, the growth pattern of cells began to display an exponential growth phase. On the days 6, the cells reached to the plateau phase. There was no significant differences between two groups (*t* test.).

Based on the CCK-8 analysis result, cells of GARP-sh group and NC group were in the slow growth period on the first two days after seeding in the plate. On the days 3, the growth pattern of cells began to display an exponential growth phase. On the days 6, the cells reached to the plateau phase (Fig. 8). Compared to the NC group, No significant difference was showed and the silence of GARP didn’t affect the viability and proliferation of BMSCs.

### Decreased levels of active TGF*β*1 were detected after transfection.

**Figure 9:**
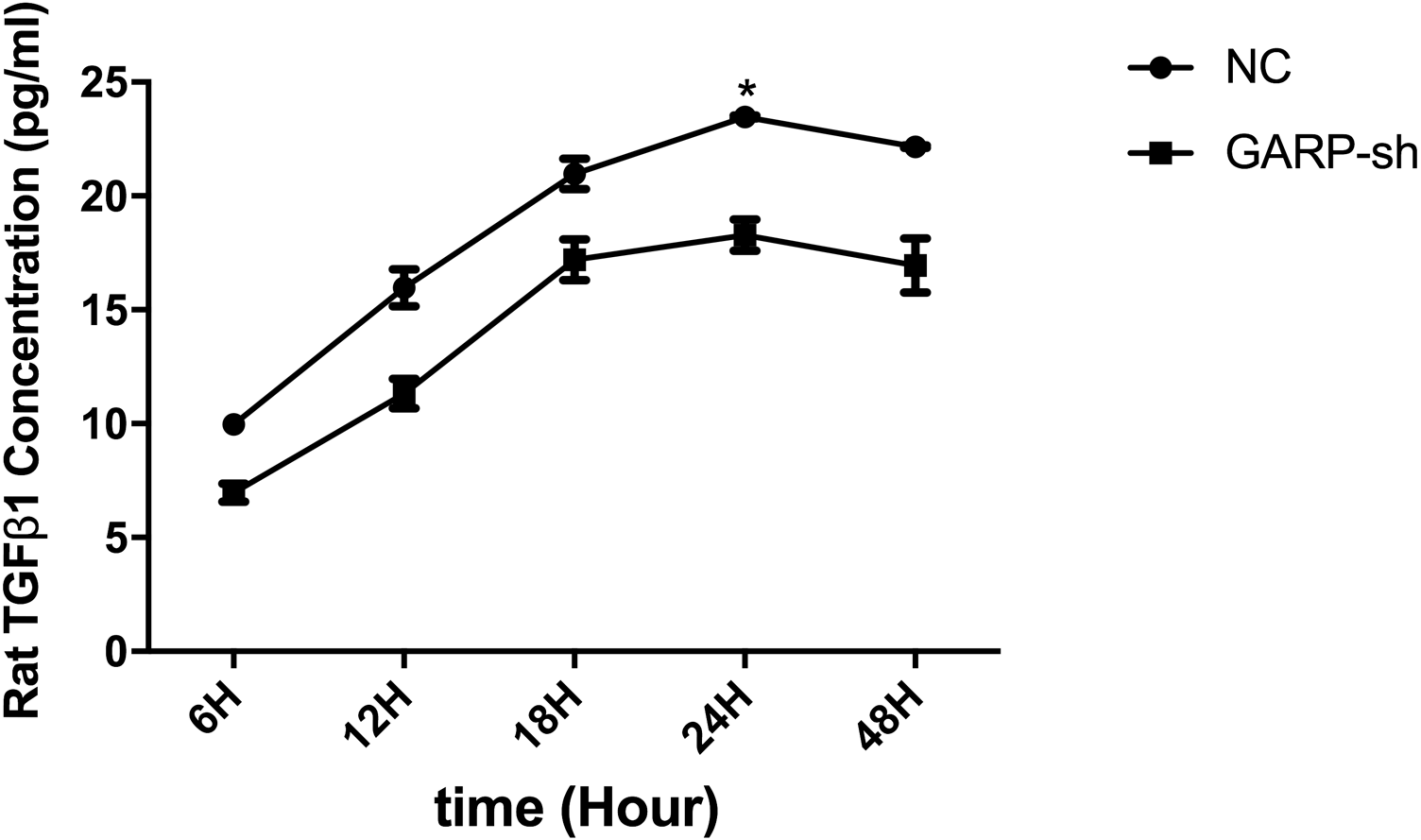
ELISA experiments of mature TGF*β*1 level in GARP-sh and NC groups. ELISA experiments showed that mature TGFβ1 level was down-regulated in GARP-sh group.(*t* test, * *P* < 0.05).

It was reported that GARP bound latent TGFβ1 on the cell surface (Tran et al., 2009), so we proposed that a decreased GARP protein level may affect TGF β1 secretion. To confirm this possibility, we developed an ELISA to determine the total TGFβ1 expression of supernatants from the control and GARP-sh groups. We collected supernatants at 6 h, 12 h, 24 h, and 48 h after the change of fresh medium. The results showed that the TGFβ1 secretion level was gradually increased before 24 h and then reached a plateau (Fig. 9). Based on comparison of the two groups, silencing of GARP expression decreased the extracellular secretion of active TGFβ1.

### Inhibition of osteogenic differentiation of BMSCs after transfection with the GARP-sh lentivirus

Numerous factors were involved during bone ossification, and it was confirmed that ALP, Runx2, and OPN were essential to this process. A high concentration of ALP denoted the start of ossification. Runx2 is the main osteogenic differentiation component of BMSCs. OPN participates in the proliferation and calcification of BMSCs. Thus, to evaluate the osteogenic differentiation ability of BMSCs, we performed ALP activity staining, RT-qPCR, and western blotting to analyze ALP, Runx2, and the OPN protein expression levels, respectively, at different stages of osteogenic differentiation on days 0, 7, and 14.

**Figure 10:**
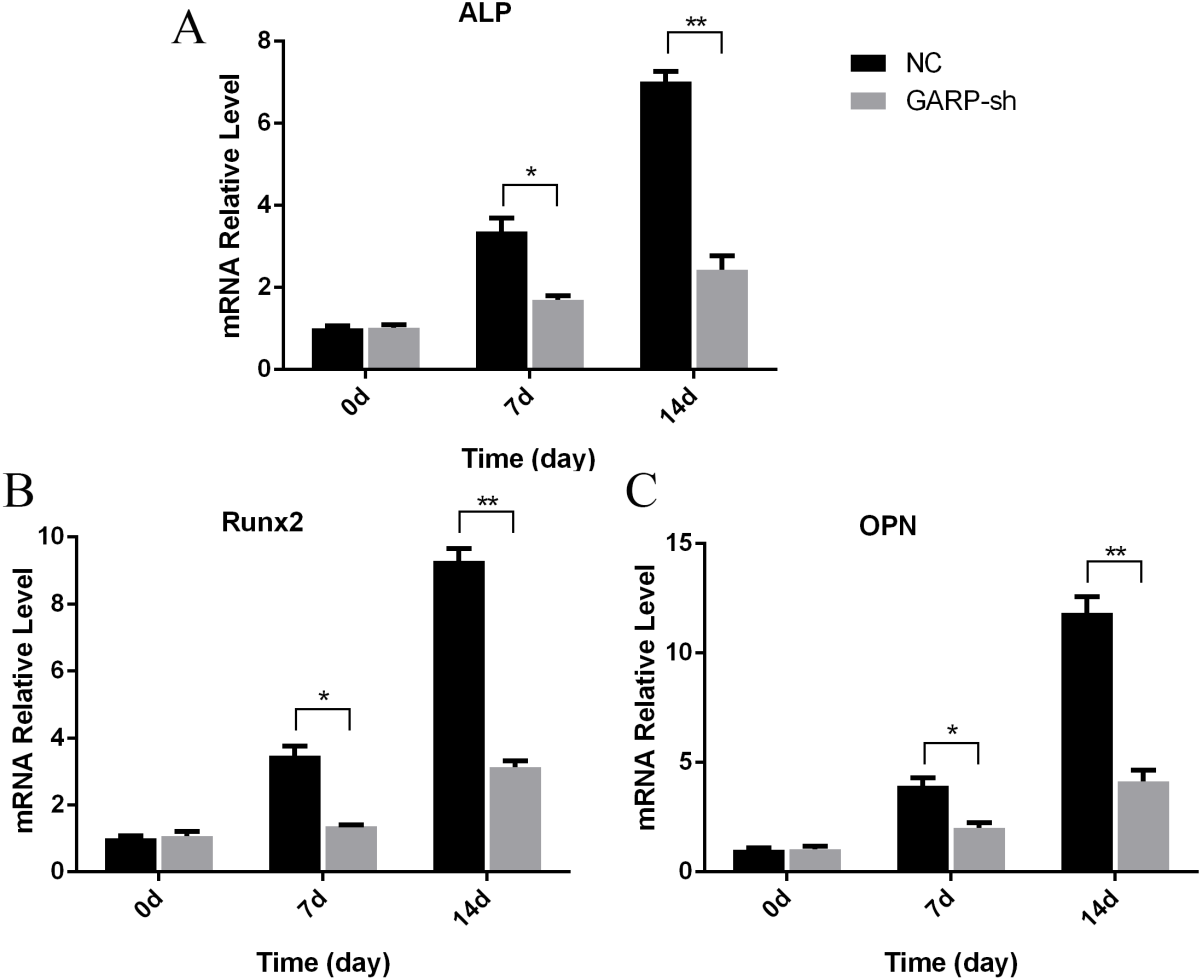
Osteogenic differentiation related factors’ mRNA expression results. Osteogenic differentiation results demonstrated ALP, Runx 2 and OPN mRNA expression was decreased in GARP-sh group at osteogenic differentiation 7 days and 14 days. (*t* test, * *P* < 0.05); (A) mRNA expression of ALP; (B) mRNA expression of Runx2; (C) mRNA expression of OPN.

RT-qPCR results showed that ALP, Runx2, and OPN expressions showed no difference on days 0 of differentiation. But on days 7, the expressions of these factors increased to varying degrees, when compared with those on days 0. In addition, the expressions of these factors in the GARP-sh group were lower than the NC group. On days 14, ALP, Runx2, and OPN expressions were higher than on days 0 and days 7, and there was a significant decline of these factors in the GARP-sh group (Fig. 10).

**Figure 11:**
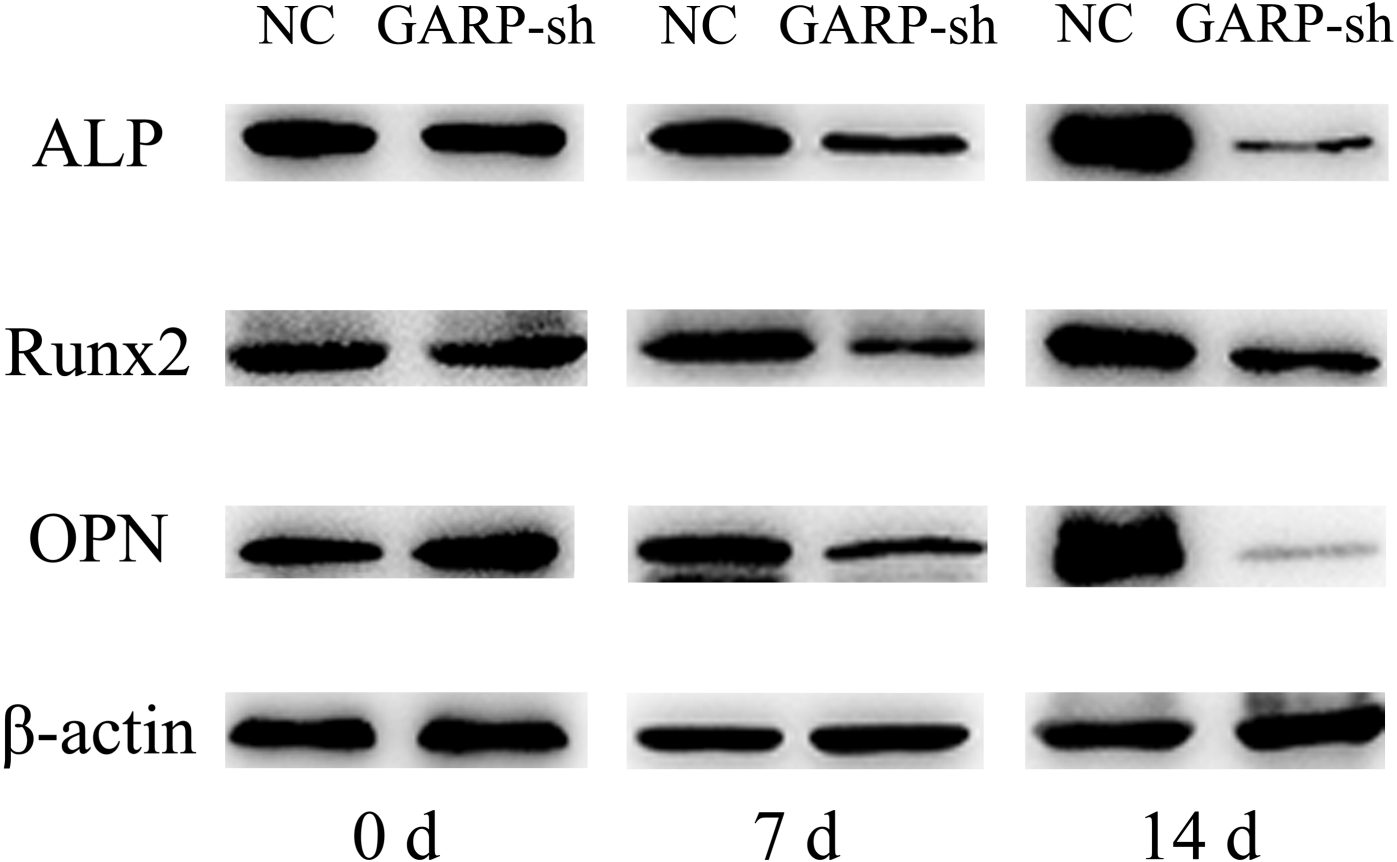
Western blot analysis of osteogenic related factors’ protein expression. Western blot analysis showed that ALP, Runx2 and OPN protein expressions were decreased in GARP-sh group.

Western blot analysis indicated that ALP, Runx2, and OPN protein levels showed no significant difference between the two groups during the pre-induction of osteogenesis. On days 7 and 14, the results displayed remarkable differences in the ALP, Runx2, and OPN expression levels between the GARP-sh and NC groups (Fig. 11).

**Figure 12:**
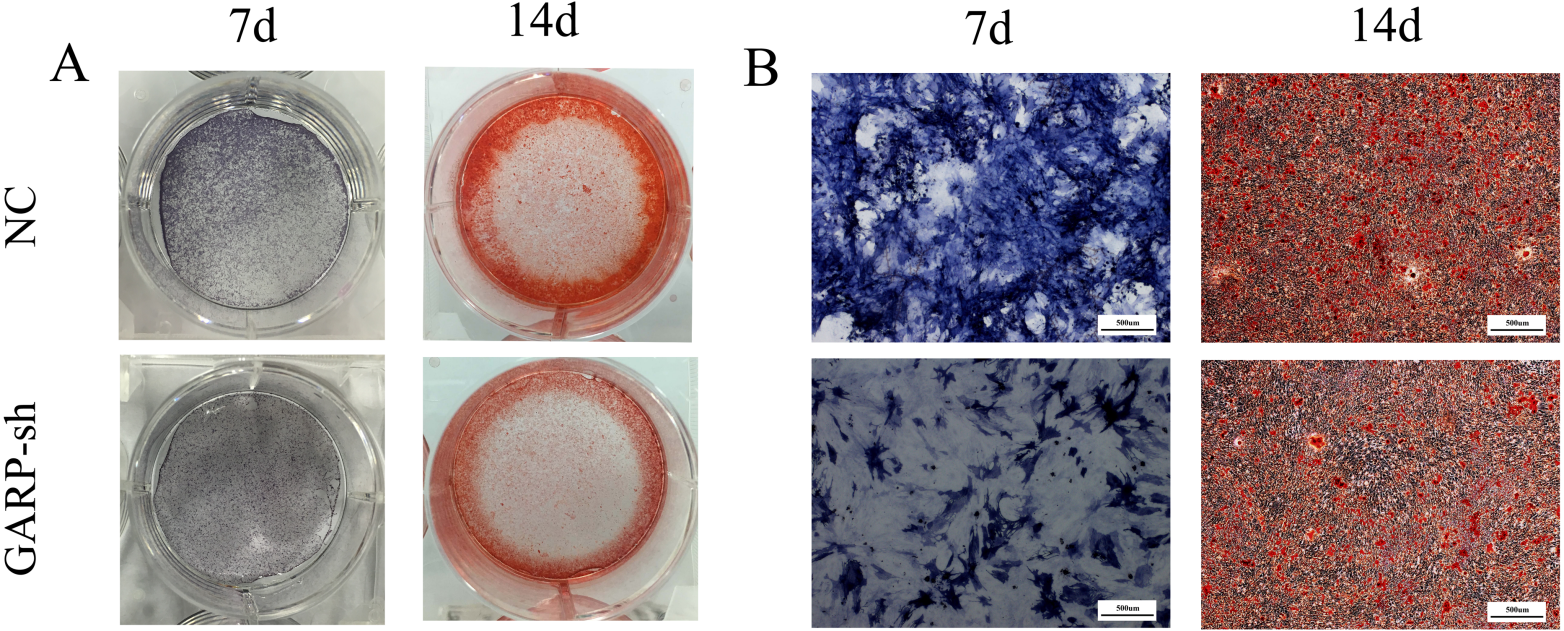
ALP and Alizarin Red S staining analysis of osteogenic differentiation. After 7 days osteogenic differentiation, ALP staining experiments showed that the staining of NC was much higher than the GARP-sh group. on 14 days after osteogenic differentiation, Alizarin Red S staining results showed that there was more mineralized nodule formation in the NC group, compared to the GARP-sh group. (A) ALP and Alizarin Red S staining with the gross appearance. (B) ALP and Alizarin Red S staining under the microscopic view (40X). Bar = 500 µm.

On the days 7, the ALP activity staining assay showed that the color of ALP staining was much more obvious than that of the GARP-sh group. On days 14, the Alizarin Red S staining level of NC group was much darker than GARP-sh group. And there are more mineralized nodule formation in the NC group, compared to the GARP-sh group (Fig. 12). Together, the results showed that ALP levels and mineralizing ability were decreased in the GARP-sh group during osteogenic differentiation.

## Discussion

BMSCs show a multilineage potential, allowing differentiation into osteoblasts, chondrocytes, adipocytes, and other tissue cells, which are easily isolated and cultured. Thus, BMSCs could be suitable cells for bone tissue engineering as osteogenic stem cells (Cancedda et al., 2000). The osteogenic differentiation process is activated by a series of signal transduction processes, of which the most important is the TGFβ pathway (Jian et al., 2006). Regulation of TGFβ may therefore provide a novel approach for the differentiation of BMSCs.

GARP, which has been mostly reported in the study of activated Treg cells, is expressed on the cell surface of activated functional FOXP3^+^ Tregs, and functions as a receptor for latent TGFβ1. Binding of TGFβ1 to the membrane is essential for the functioning of Tregs (Tran et al., 2009). With the assistance of GARP, latent TGFβ1 is localized on the cell membrane, and is then ready to release its active form (Stockis et al., 2009). However, the exact mechanism of activation of membrane latent TGFβ1 in Tregs is not known. GARP mRNA expression has been recently detected in MSCs (Barbet et al., 2011). The interaction between GARP and latent TGFβ1 has also been described in a follow-up report (Carrillo-Galvez et al., 2015). In the present study, we conducted further studies to determine the effect of GARP on the osteogenic development of BMSCs via the regulation of latent TGFβ1.

Our study determined the existence of GARP in BMSCs. To further investigate the bioactivity of GARP, we performed transfection experiments to silence the expression of GARP. The results showed that both total and membrane GARP protein expressioxns were knocked down. Furthermore, we showed that the secretion of TGFβ1 was decreased with the reduction of GARP. Previous studies have reported that TGFβ1 is secreted as an inactive form (latent TGFβ1) (Annes, Munger & Rifkin, 2003), and both LTBPs and GARP bind latent TGFβ1. LTBPs bind latent TGFβ1 by disulfide bonds. In this manner, LTBPs assist the transmembrane transportation and anchorage in the ECM, of latent TGFβ1. Furthermore, the storage of latent TGFβ1 in the ECM is functionally activated under specific conditions (Taipale et al., 1994). GARP can anchor latent TGFβ1 noncovalently to the cell membrane. The membrane GARP/latent TGFβ1 complex serves as a source of active TGFβ1. The membrane latent TGFβ1 can be activated by mediation of integrins *α*v β6 (Wang et al., 2012). In our study, the decrease of membrane GARP provided an insufficient amount of latent TGFβ1, so eventually the secretion of TGFβ1 was lower than untreated BMSCs. In contrast to the results of Carrillo-Galvez et al., our data indicated that silencing of GARP resulted in the reduction of TGFβ1. It was reported that GARP competed better with LTBPs when binding to latent TGFβ1 (Wang et al., 2012). We speculated that membrane-associated GARP plays a more important role than the LTBPs in the process of TGFβ1 activation.

TGFβ1 can be produced by BMSCs and participates in osteoblast differentiation of stem cells as a pleiotropic molecule. The activation of the SMAD signaling pathway by TGFβ1 can increase the osteoblast differentiation (Jian et al., 2006; Kulterer et al., 2007; Ng et al., 2008; Miron et al., 2013). A previous study reported that TGFβ1 actions can be attributed to the foundation of bone mass and quality through the regulation of perilacunar/canalicular remodeling (Dole et al., 2017). In the present study, we showed that osteogenic-related genes of ALP, Runx2, and OPN were significantly downregulated with decreased secretion of TGFβ1, and that the suppression of osteogenic differentiation was also confirmed at the translational level. According to our western blot results, all ALP, Runx2, and OPN protein expression levels were significantly attenuated compared to the untreated group. Moreover, the ALP activity of GARP-sh cells was lower than that of untreated BMSCs. These results indicated that the decreased amount of TGFβ1 inhibited the osteogenesis of BMSCs. However, the biological mechanism remains to be definitively elucidated. Further animal experiments are necessary to demonstrate that the osteogenic differentiation is affected by the GARP/latent TGFβ1 complex *in vivo*.

It has been shown that MSCs reside in the periodontal ligament, which may be a promising reservoir for regeneration (Beertsen, McCulloch & Sodek, 1997). In addition, TGFβ1 can cooperate with other growth factors, such as bone morphogenetic proteins (BMPs), to induce osteoblast differentiation (De Gorter et al., 2011; Hyun et al., 2017). In the present study, GARP was responsible for the regulation of the activation and secretion processes of latent TGFβ1. Furthermore, GARP indirectly regulated osteogenic differentiation of BMSCs. These results may provide promising applications in the field of tissue engineering.

## Conclusions

We verified that the downregulation of GARP decreased the level of mature TGFβ1 and the osteogenic ability in rat BMSCs. However, bone regeneration is a rather complicated process that is regulated by various factors. It still remains to be elucidated whether a decrease in the levels of mature TGFβ1 reduces the osteogenic ability *in vivo*. Based on the results above, we hypothesize that the osteogenic ability can be improved by increased TGFβ1 levels via the upregulation of GARP. The regulation of GARP on levels of TGFβ1 may therefore provide a novel solution to challenges in tissue engineering and bone regeneration.

##  Supplemental Information

10.7717/peerj.6993/supp-1 Dataset S1 Raw data of BMSCs images under light microscope

10.7717/peerj.6993/supp-2 Dataset S2 Raw data of GARP expression

10.7717/peerj.6993/supp-3 Dataset S3 Raw data of osteogenic differentiation

## Additional Information and Declarations

### Data Availability

The following information was supplied regarding data availability: The raw data is available in the Supplemental Files.
