# Supplementary figures and images for: Effect of GARP on osteogenic differentiation of bone marrow mesenchymal stem cells via the regulation of TGFβ1 in vitro
Source: PeerJ. 2019 May 23;7:e6993. doi: 10.7717/peerj.6993 (PMC6535220; doi:10.7717/peerj.6993)

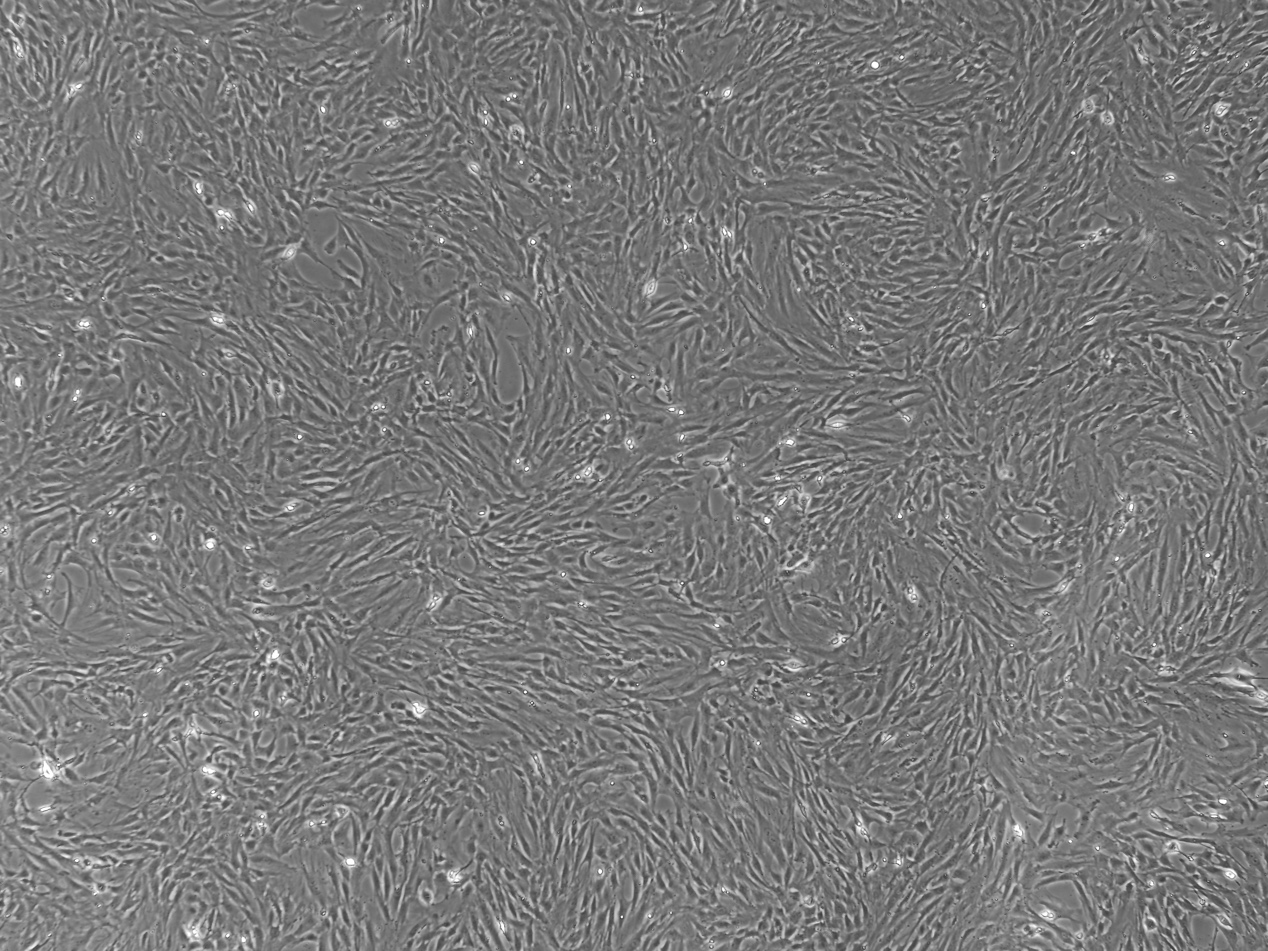


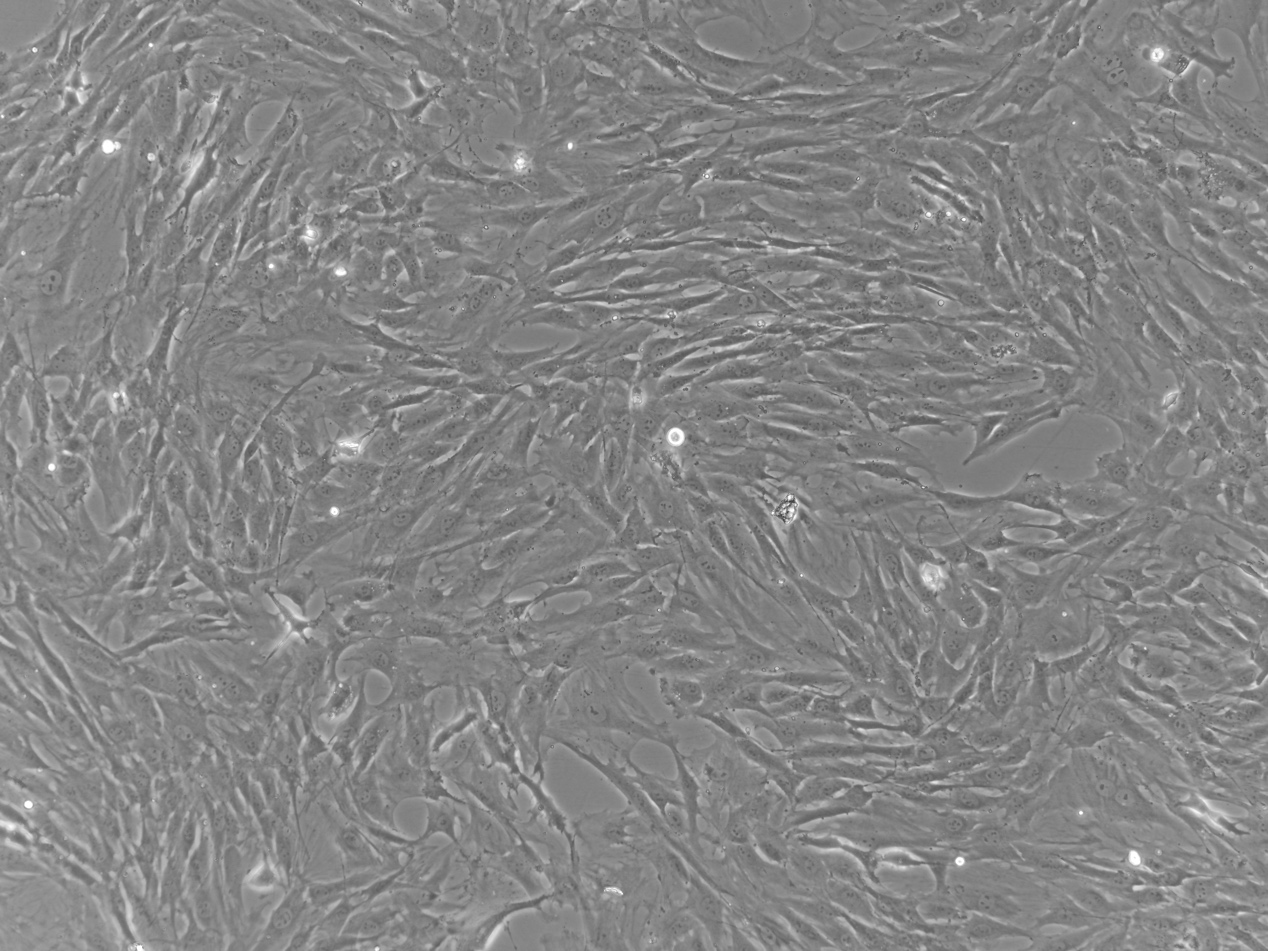


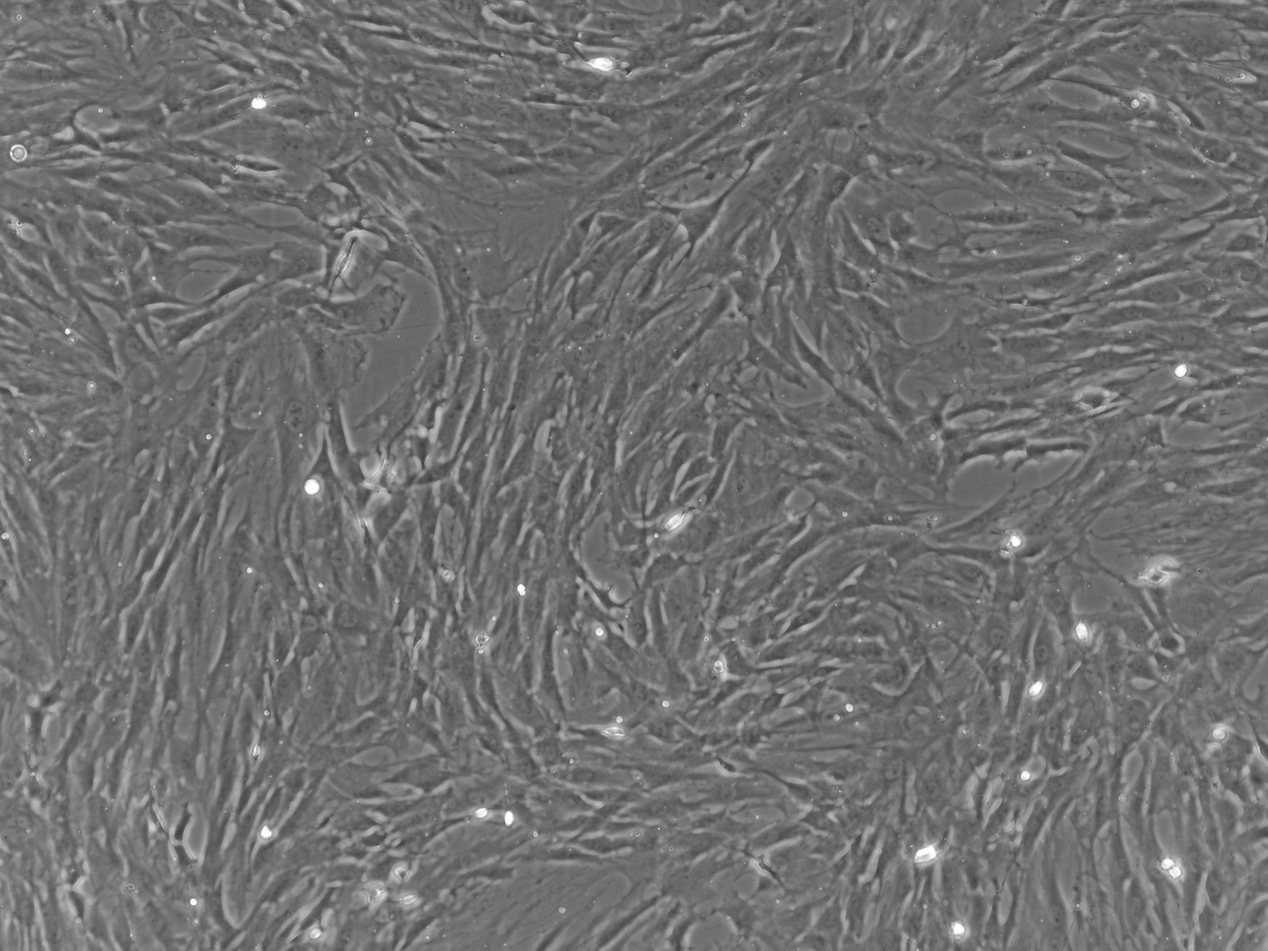


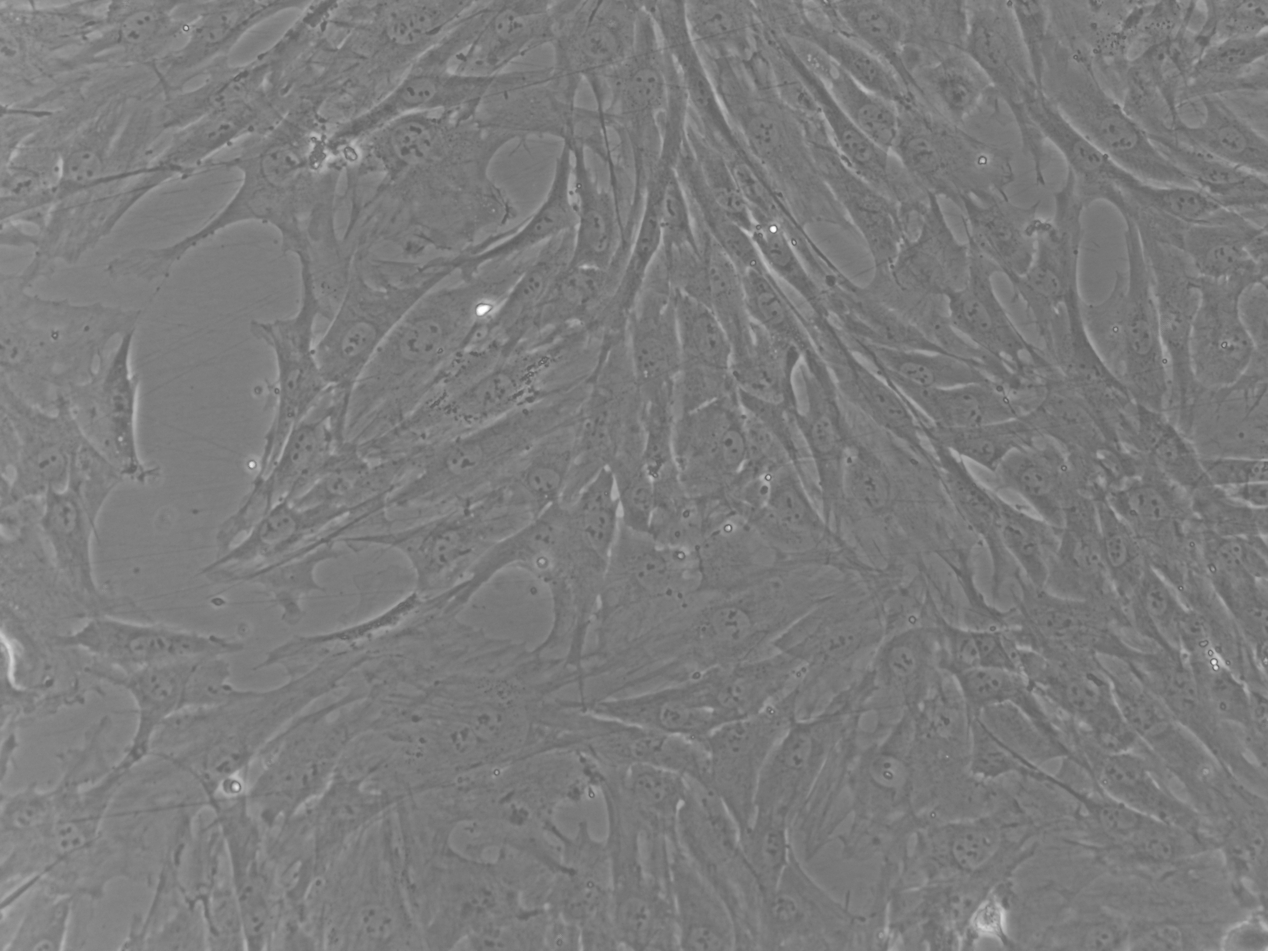


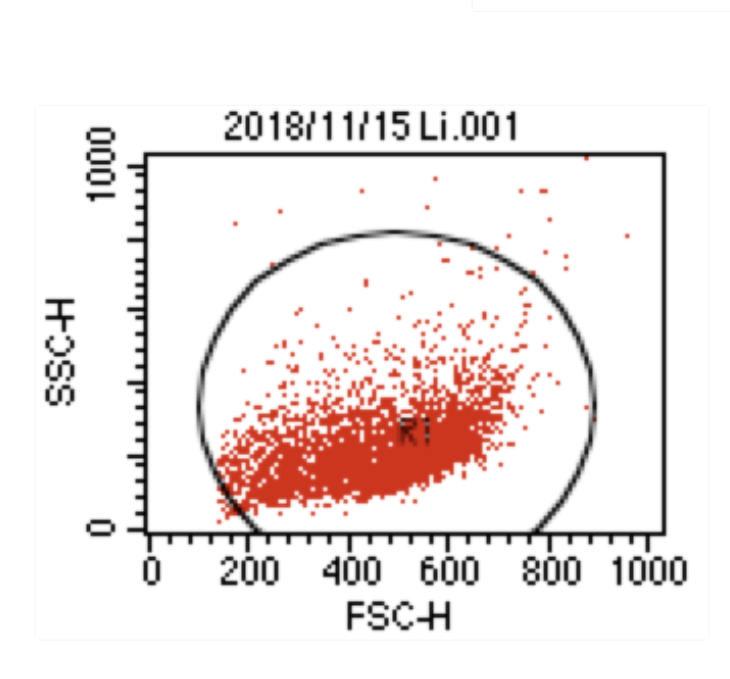


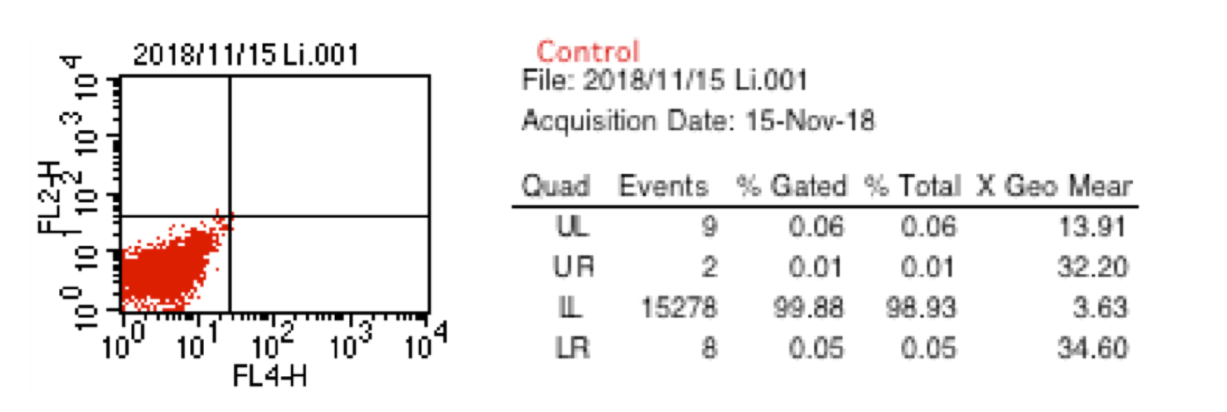

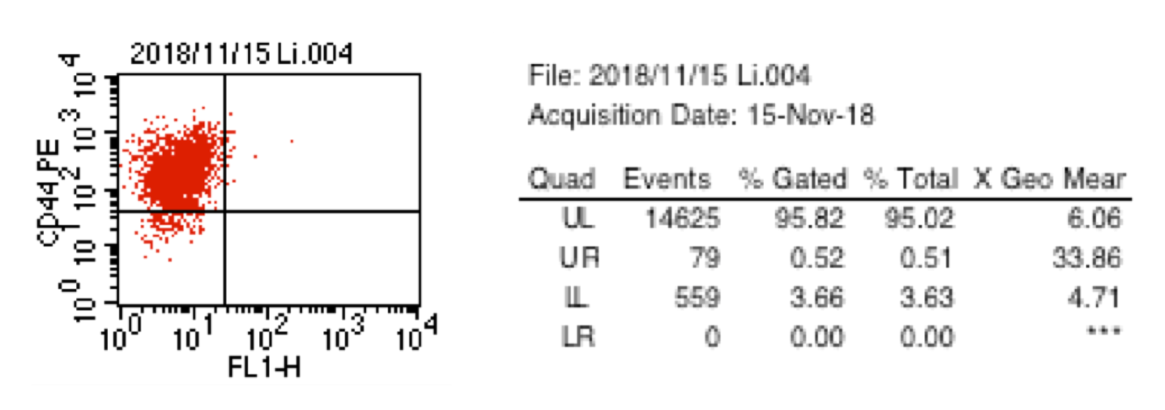


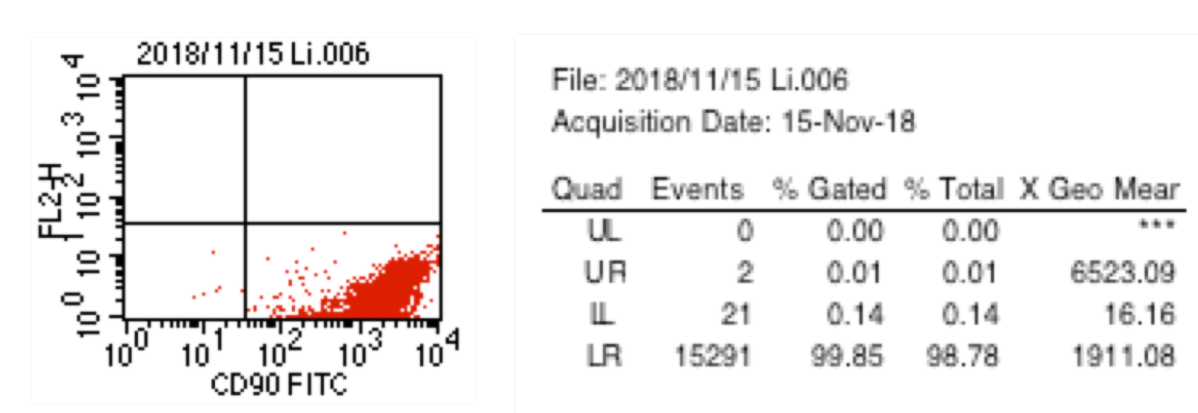


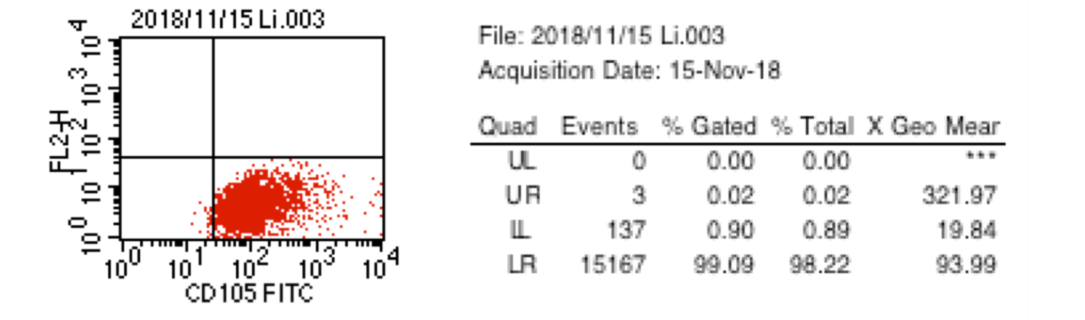


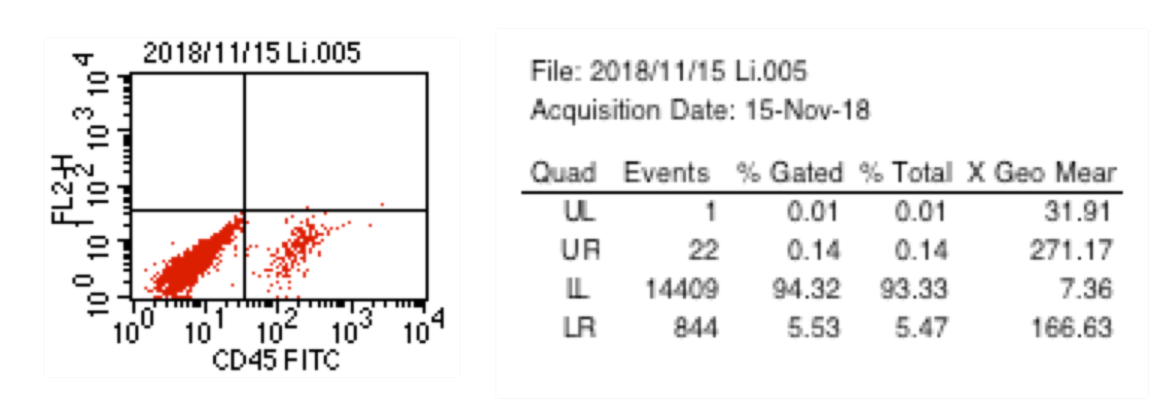

Supplement: Dataset S1 [file peerj-07-6993-s001.docx]

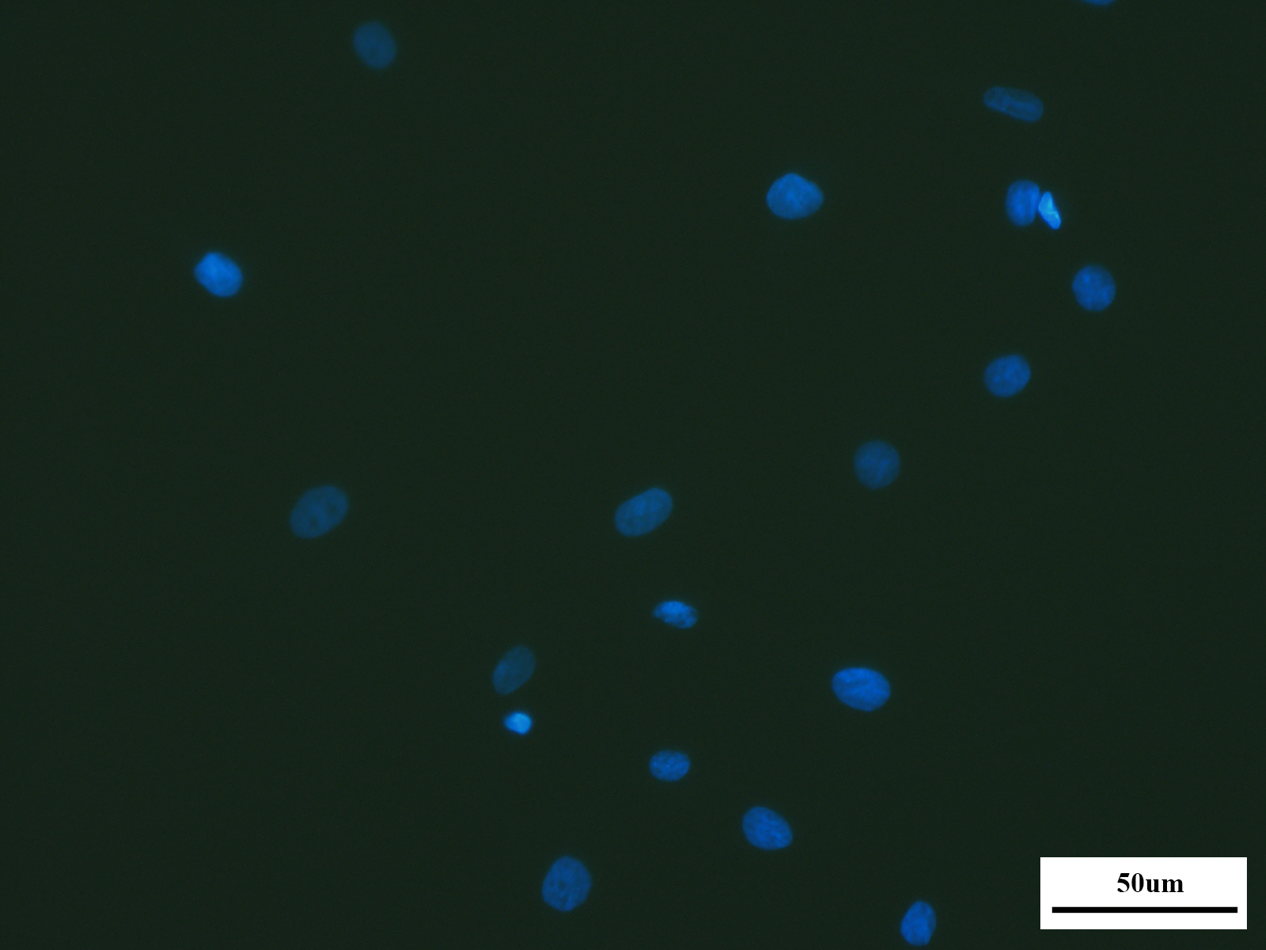


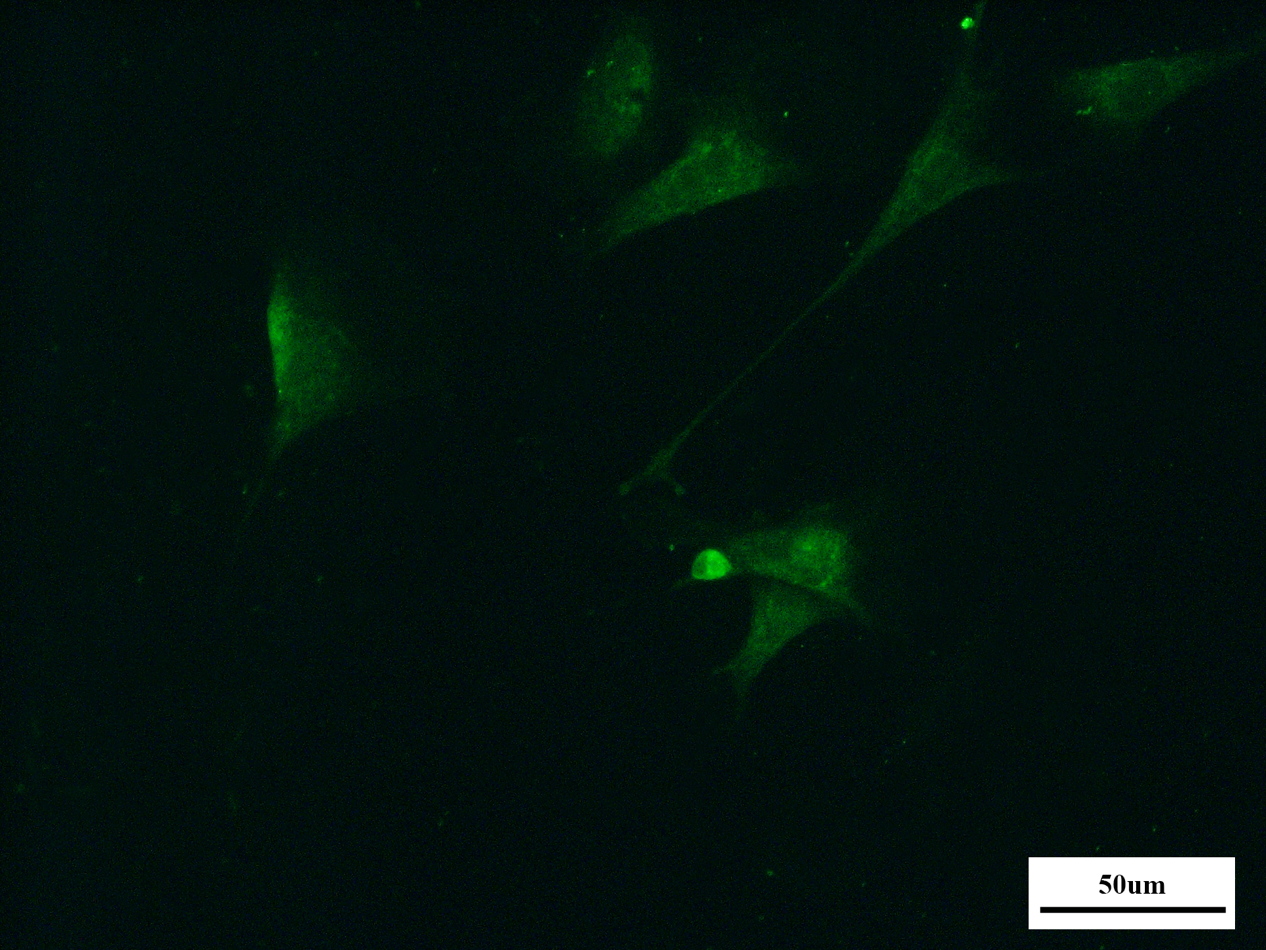

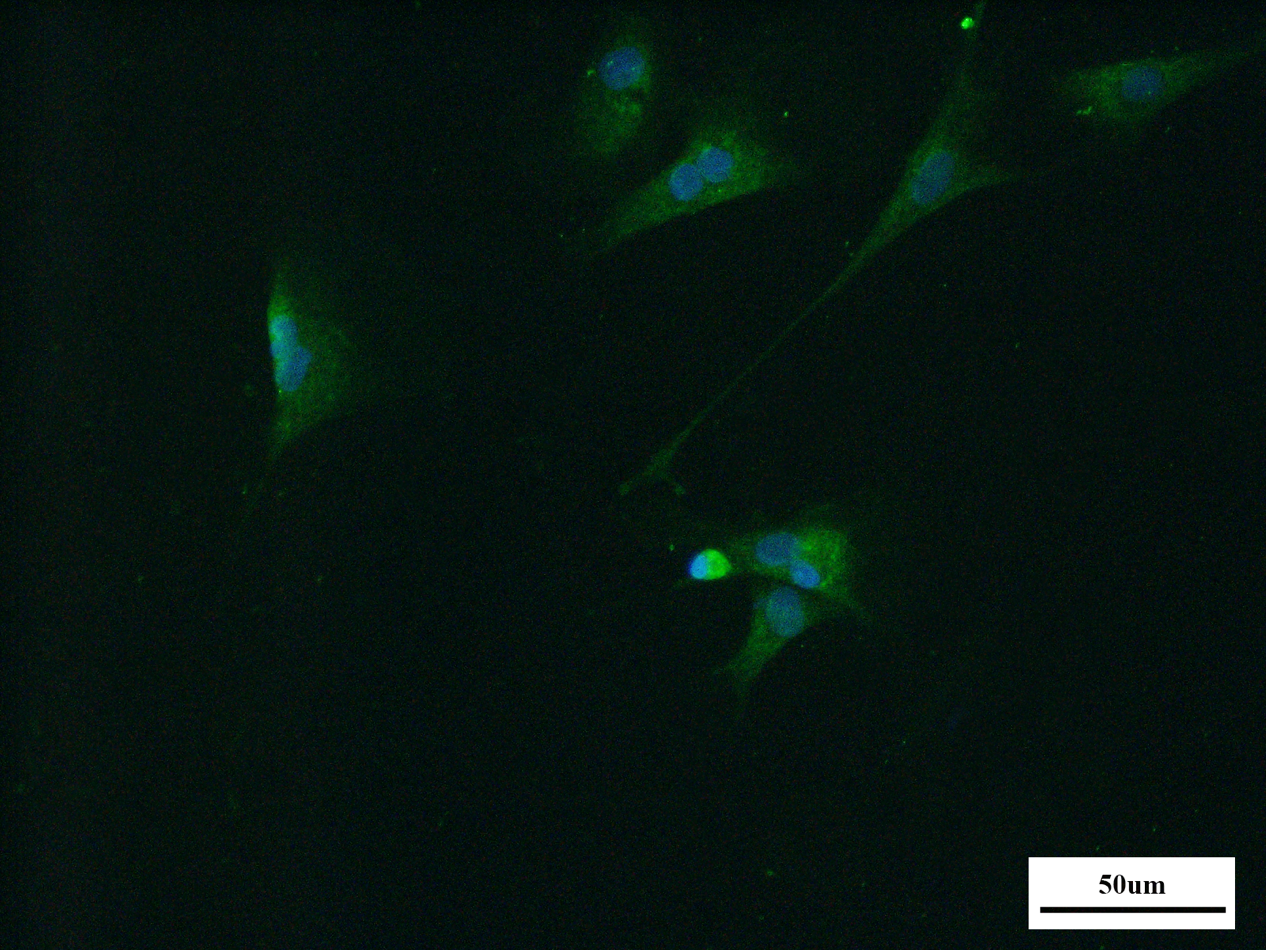


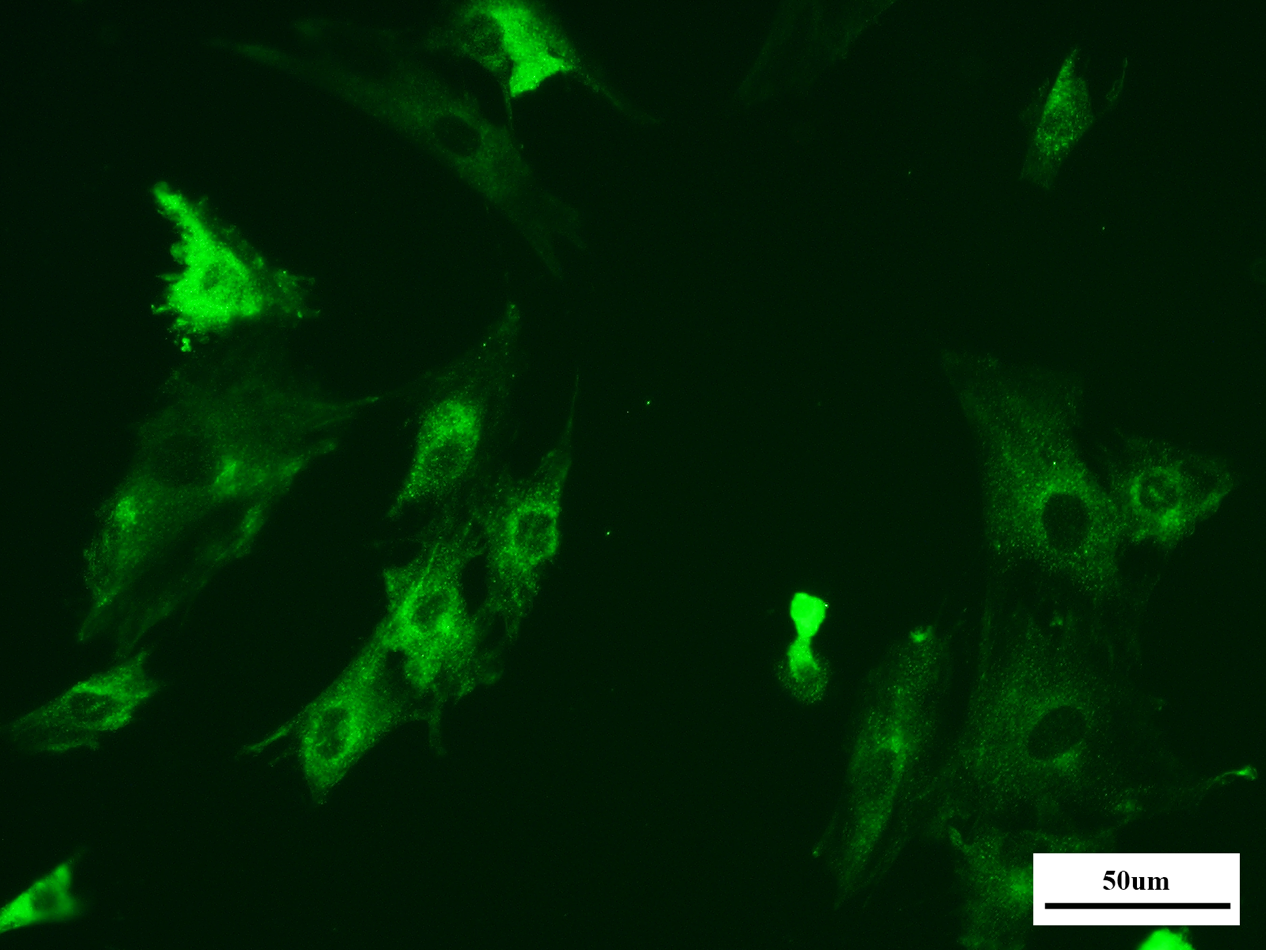


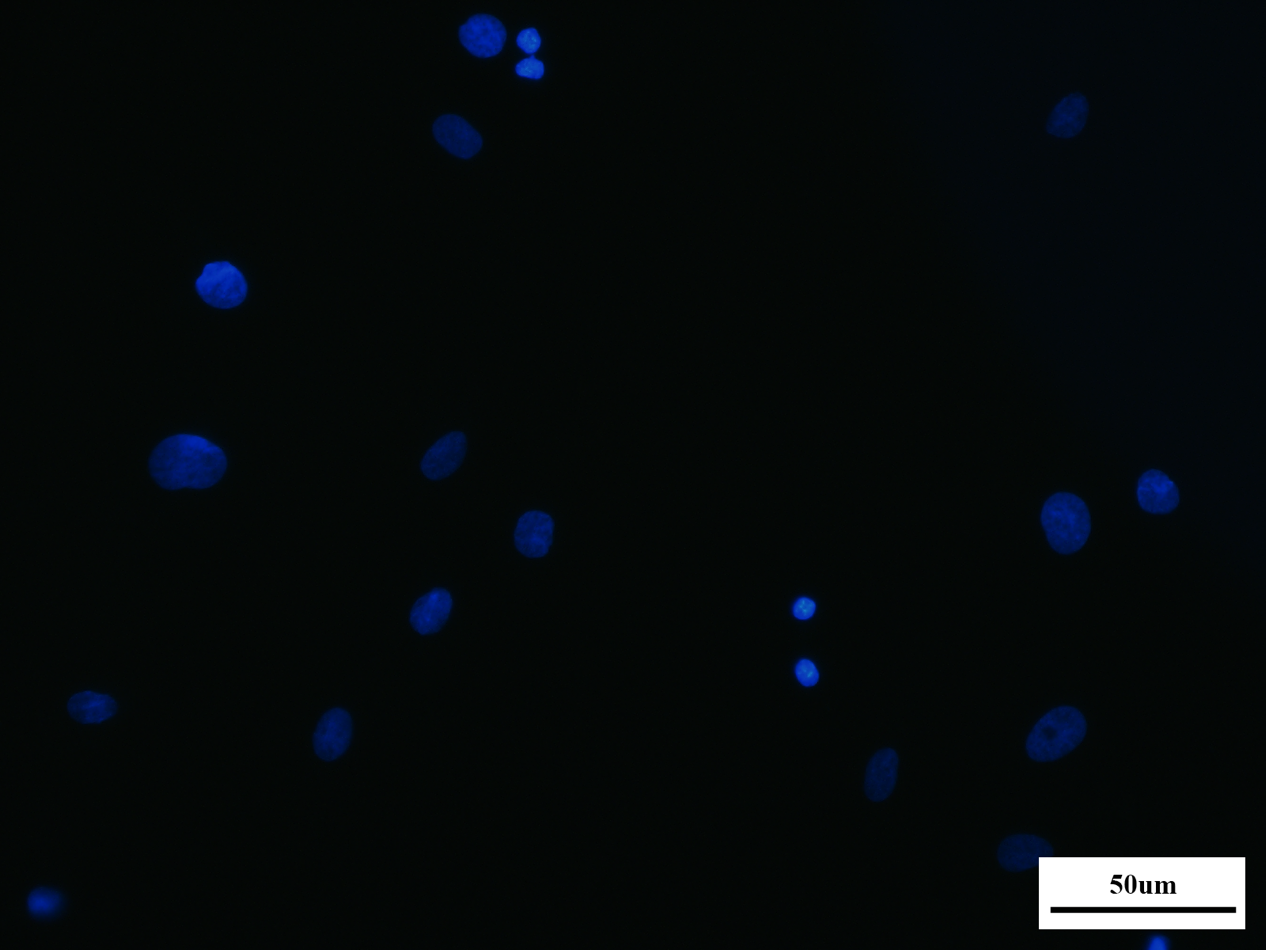


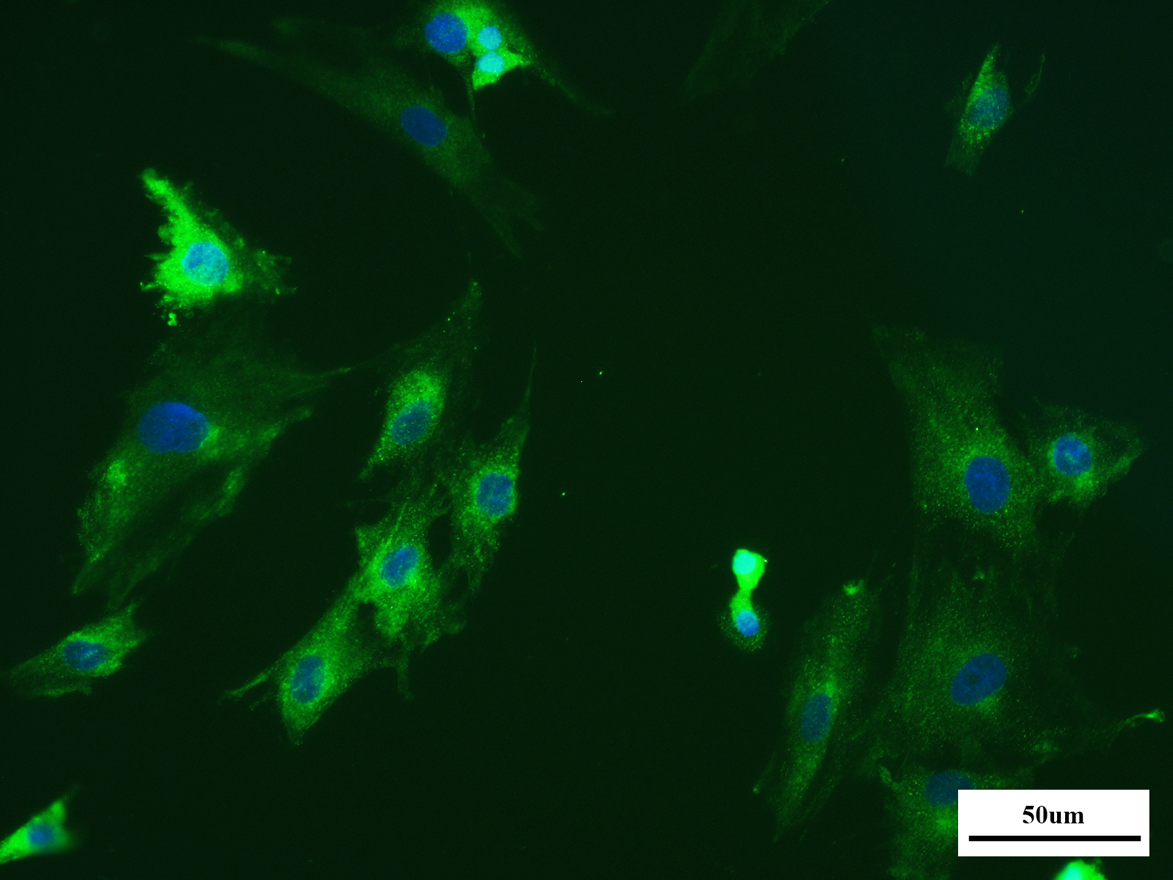


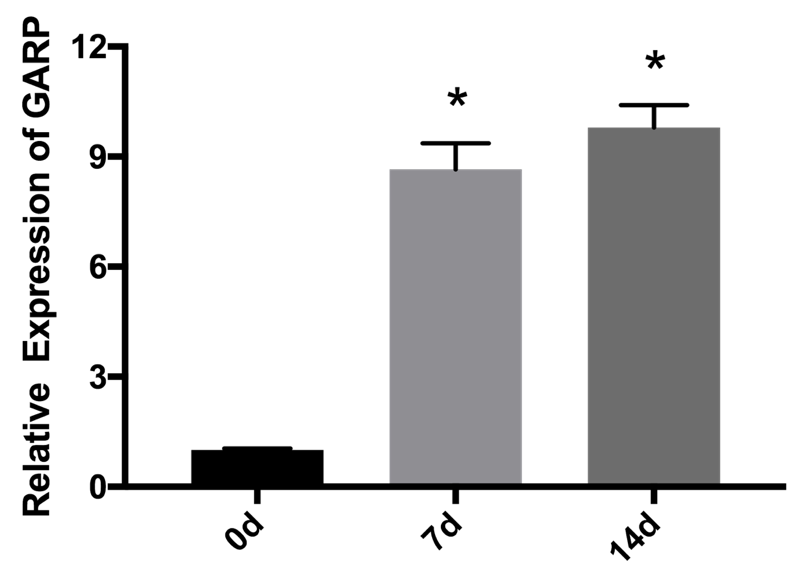


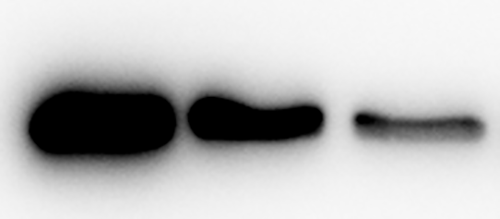


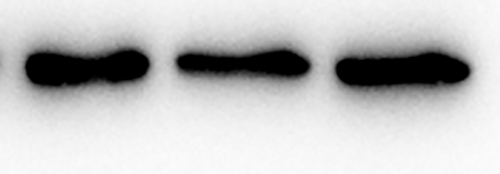


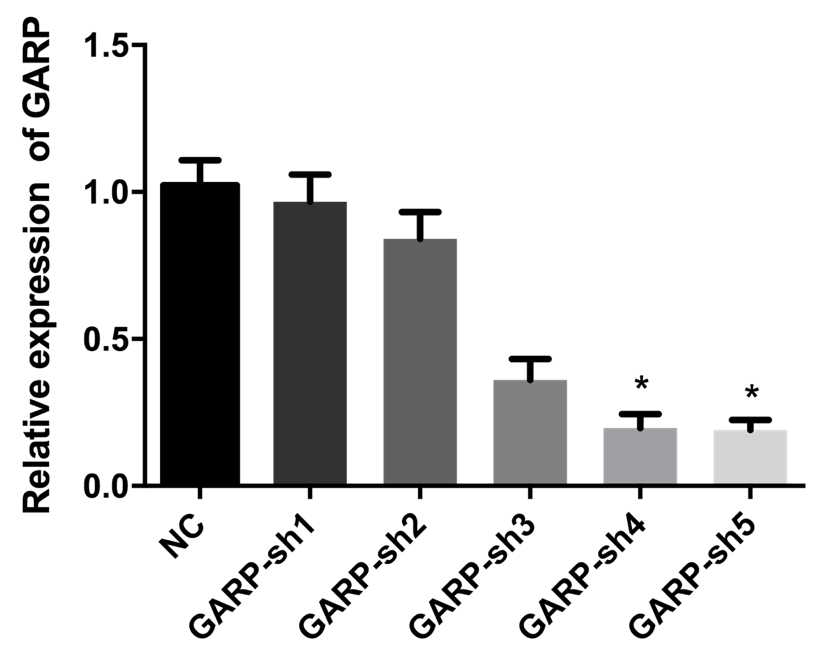


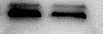


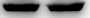


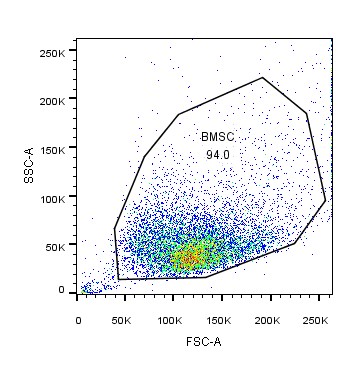


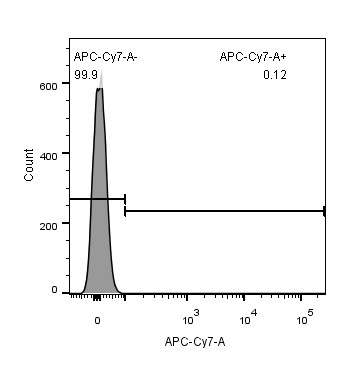


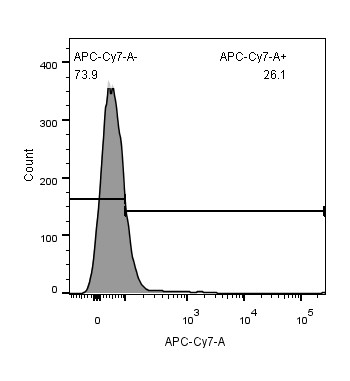


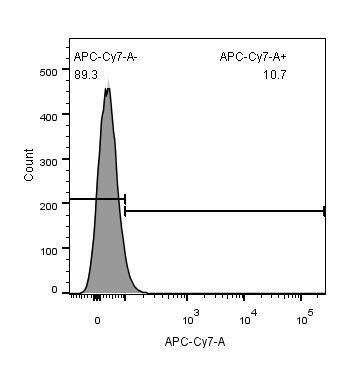

Supplement: Dataset S2 [file peerj-07-6993-s002.docx]
