# Supplementary material for: Effect of GARP on osteogenic differentiation of bone marrow mesenchymal stem cells via the regulation of TGFβ1 in vitro
Source: PeerJ. 2019 May 23;7:e6993. doi: 10.7717/peerj.6993 (PMC6535220; doi:10.7717/peerj.6993)

Raw data of osteogenic differentiation

0 d (ALP, Runx2, OPN, Actin)


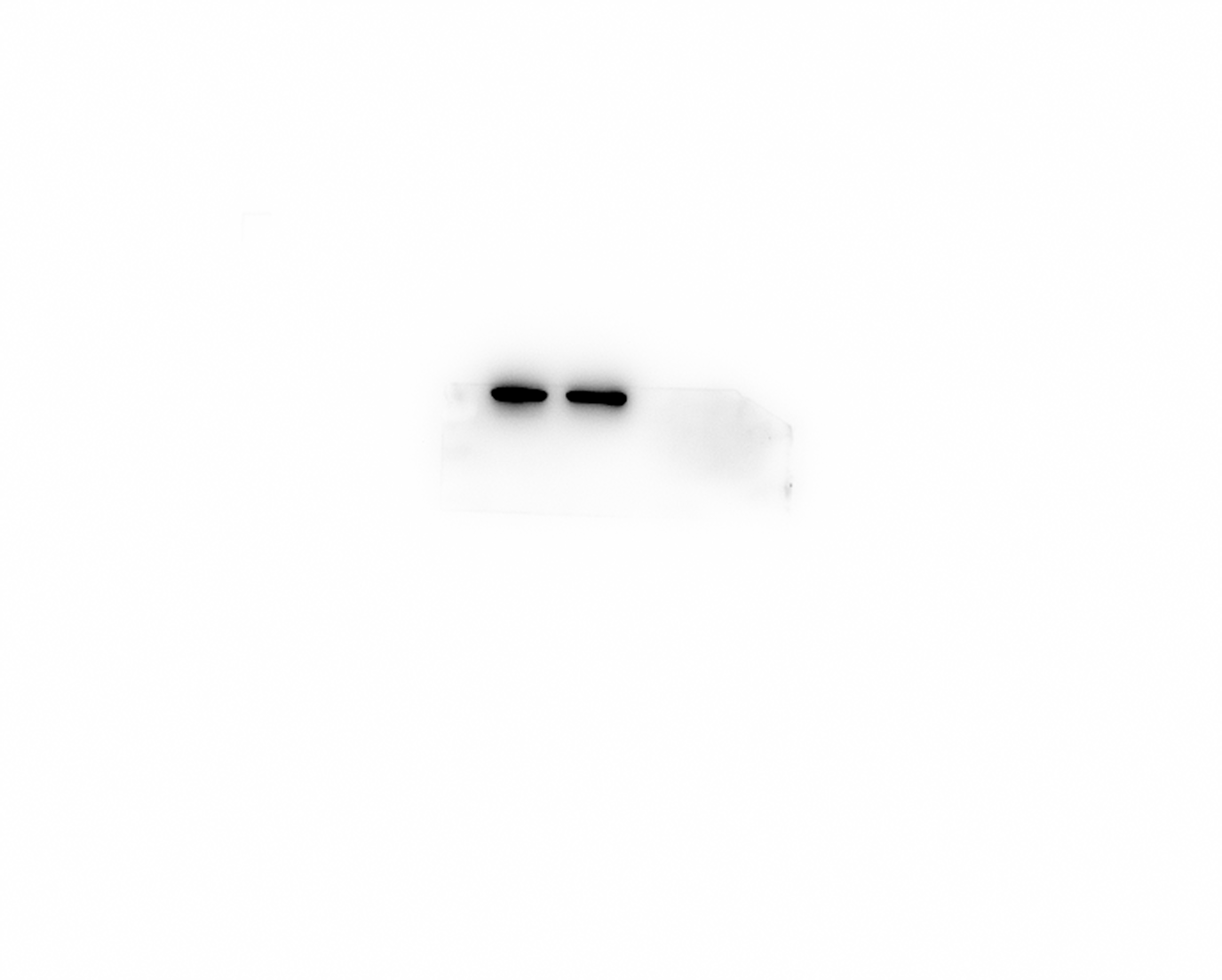


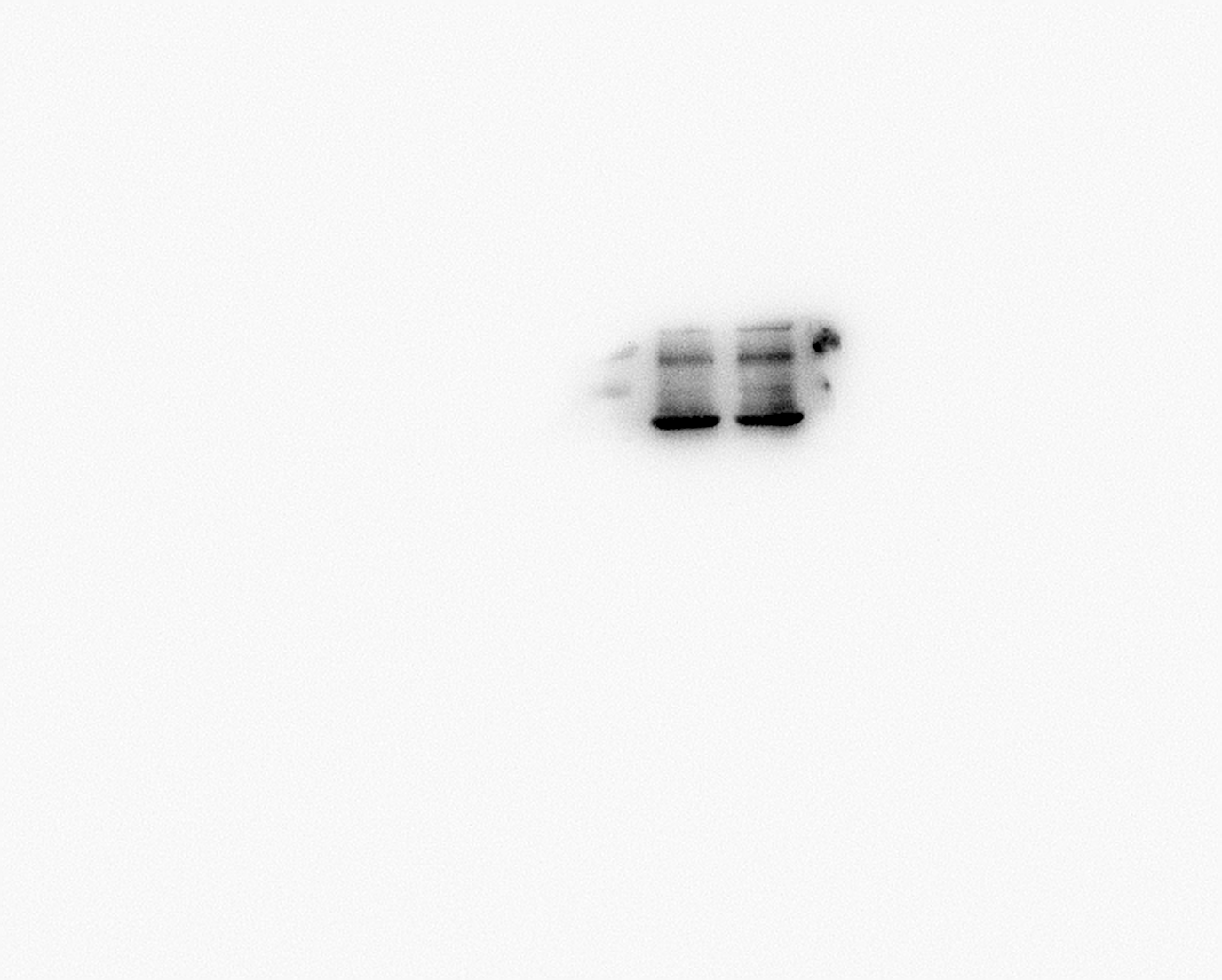


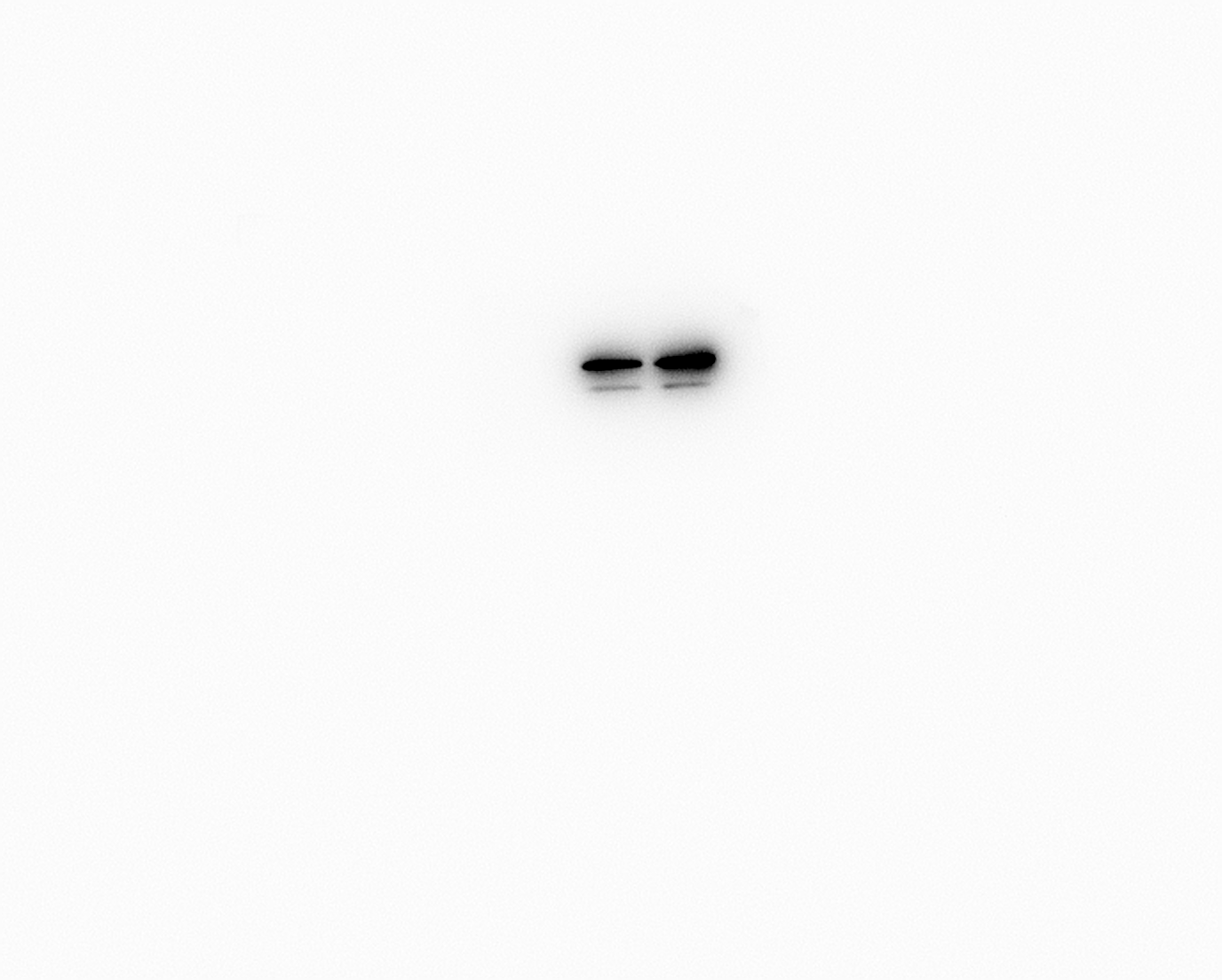


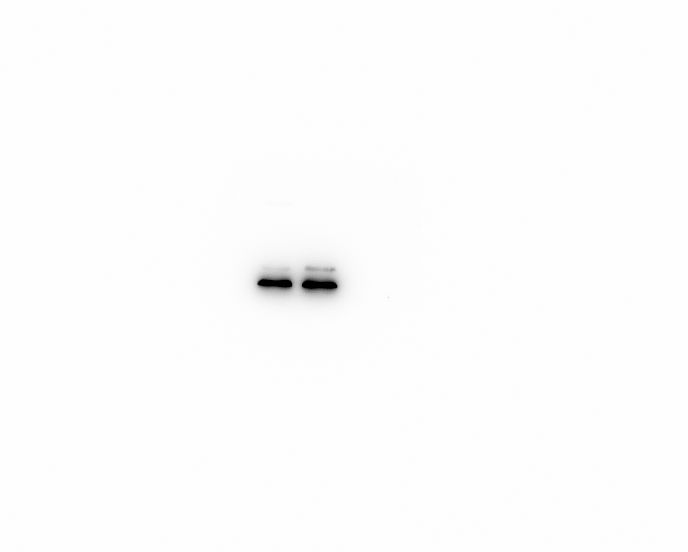


7 d (ALP, Runx2, OPN, Actin)


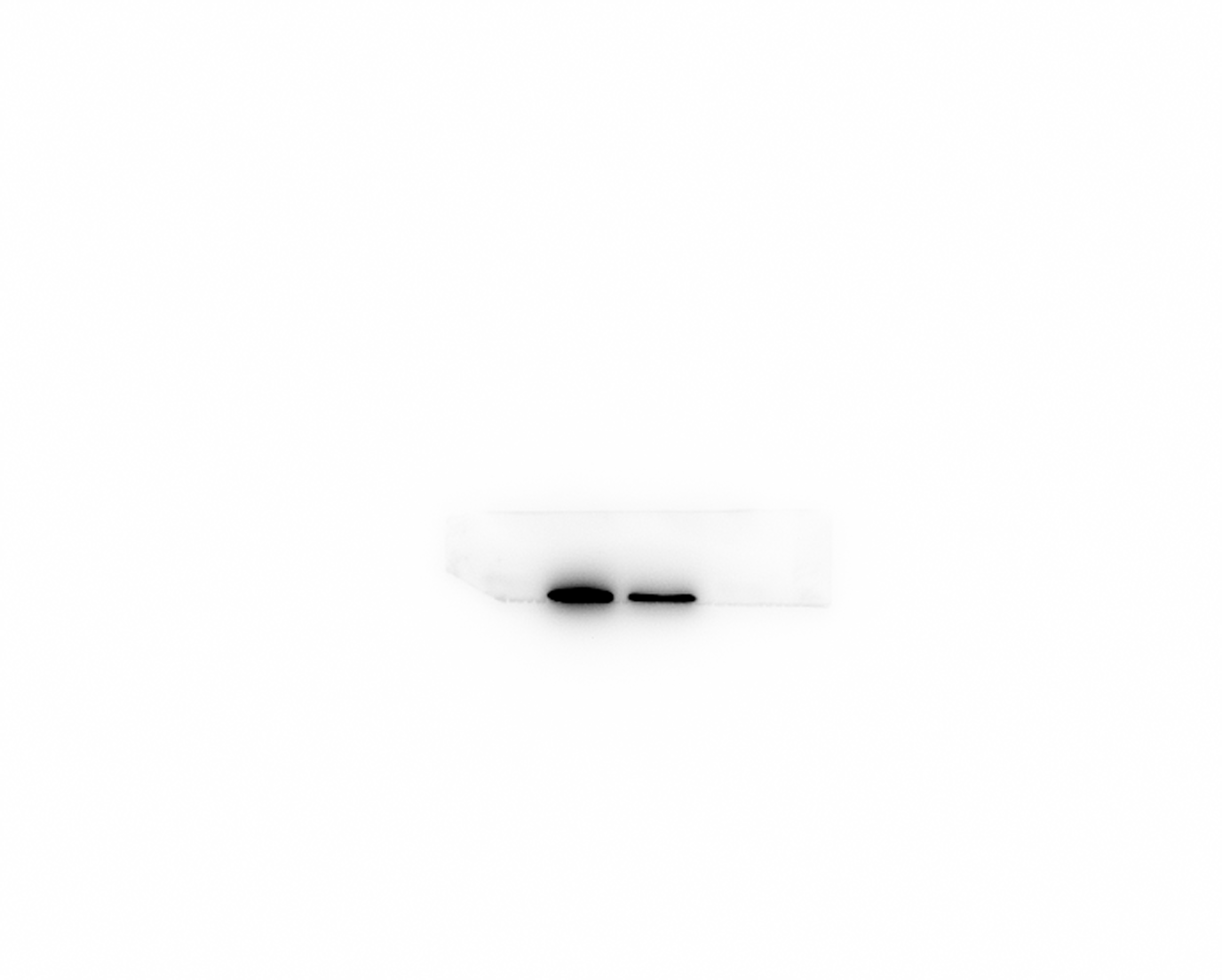


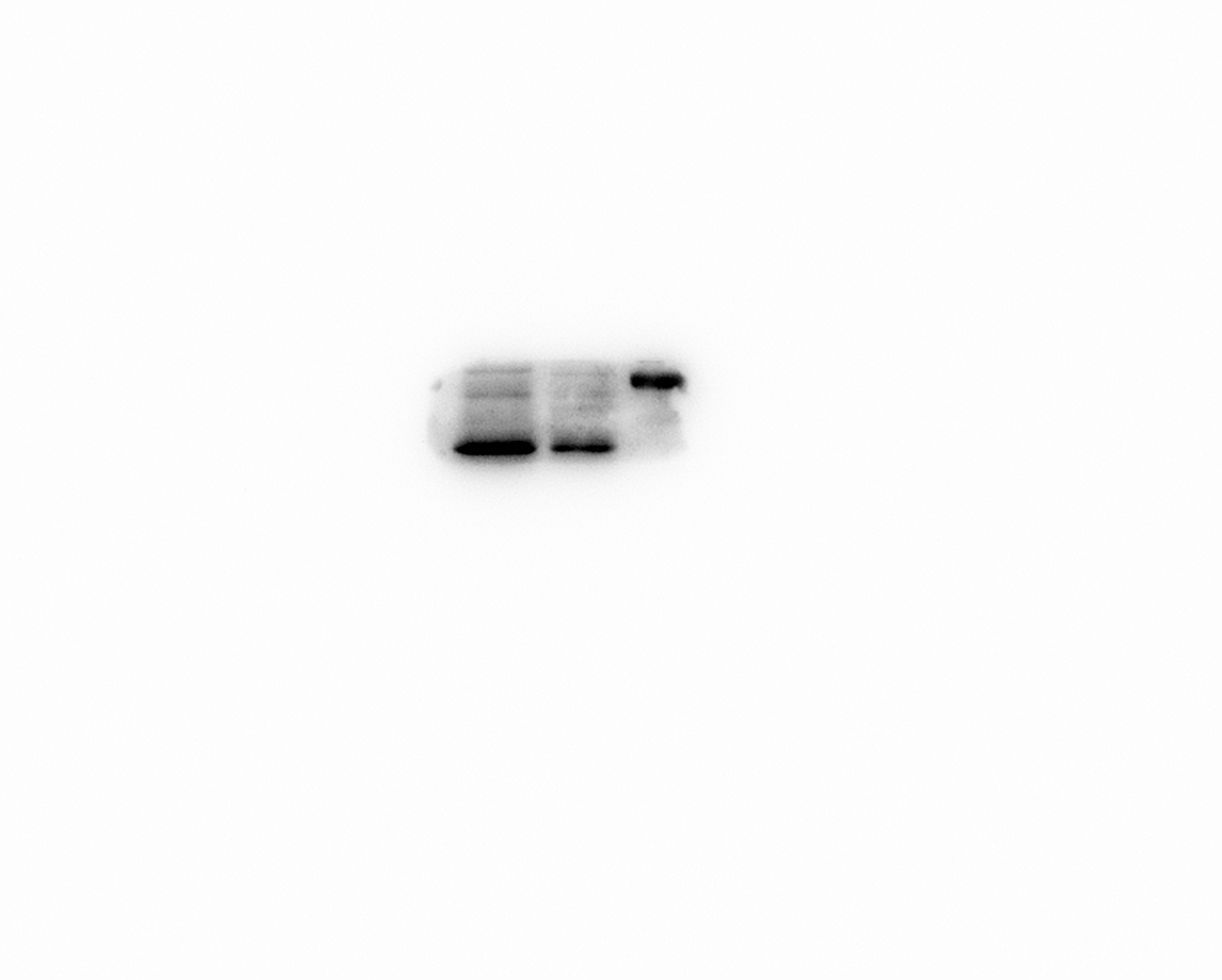


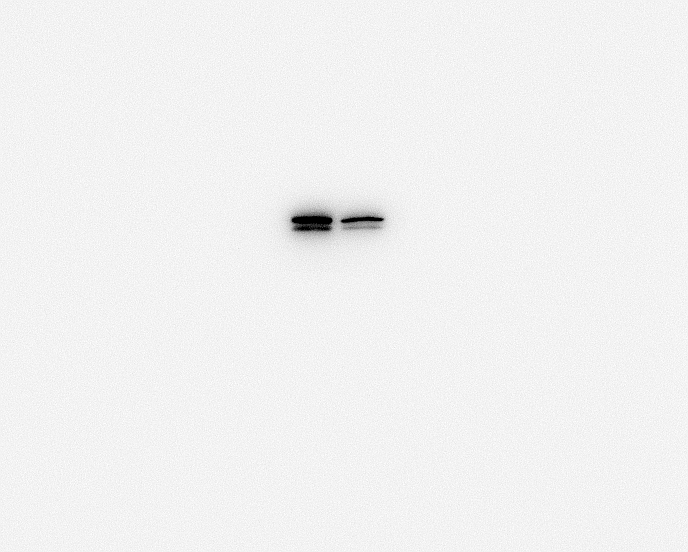


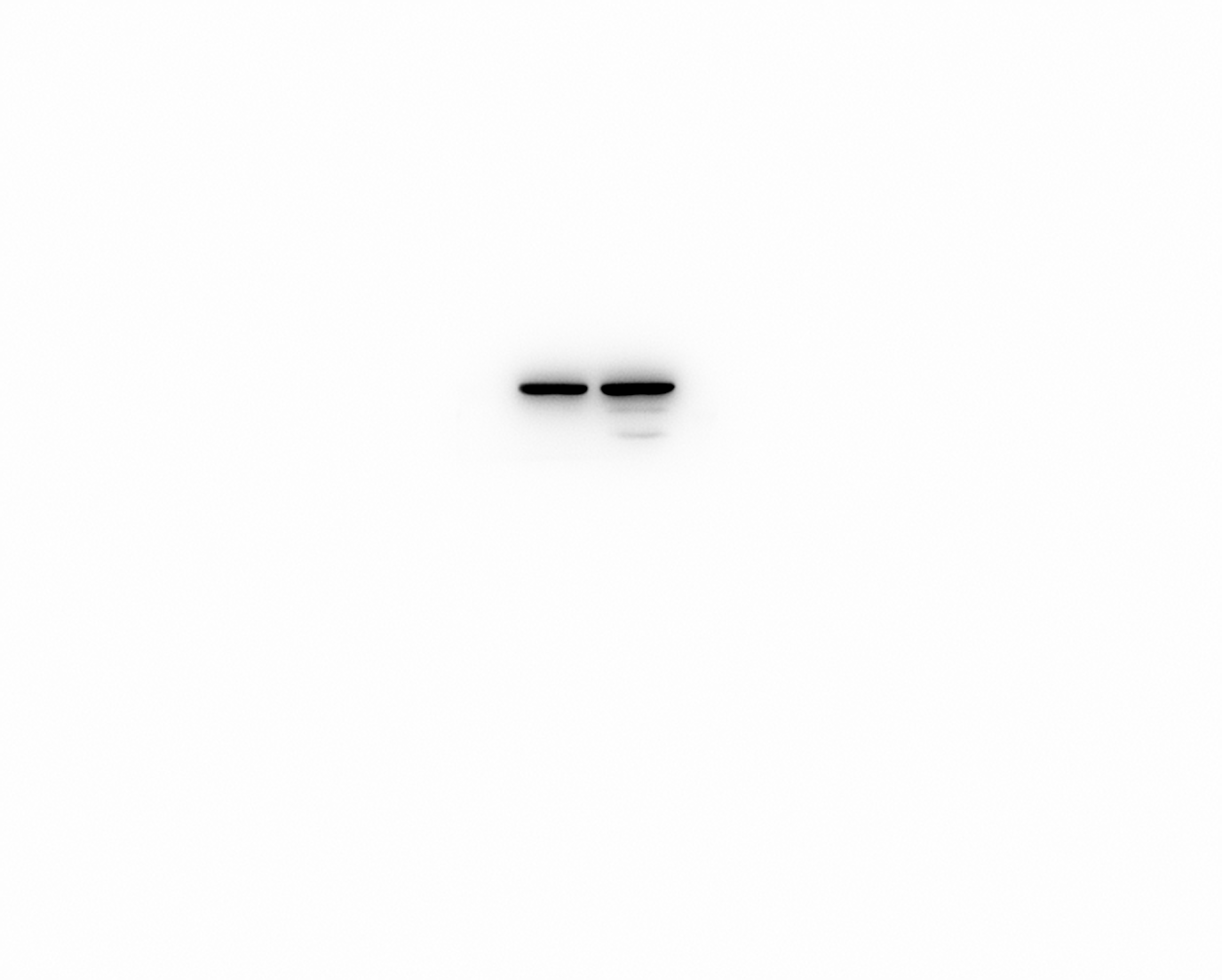


14 d (ALP, Runx2, OPN, Actin)


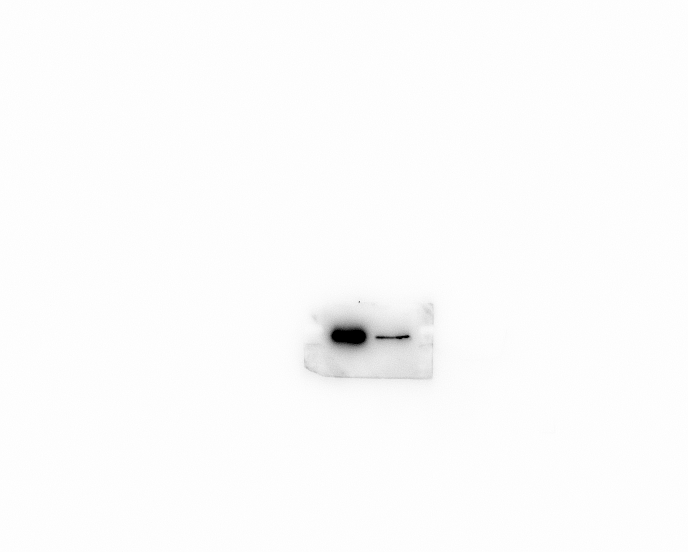


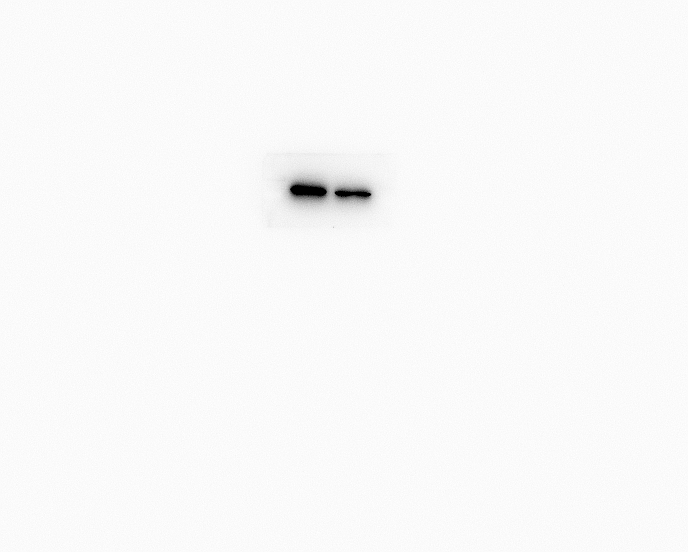


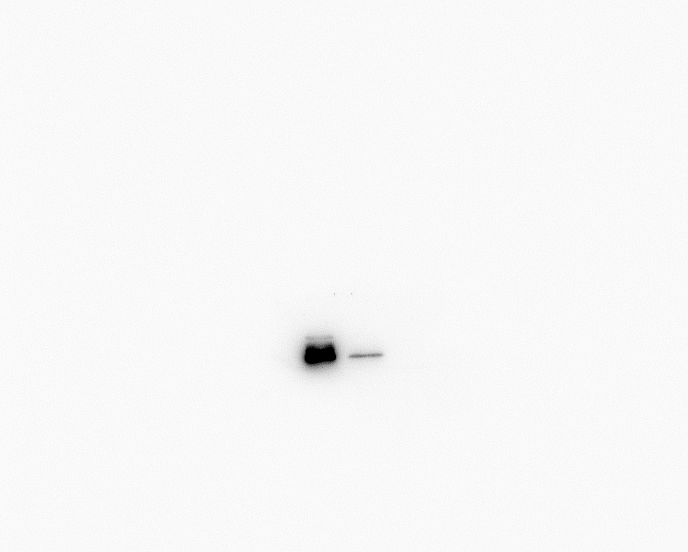


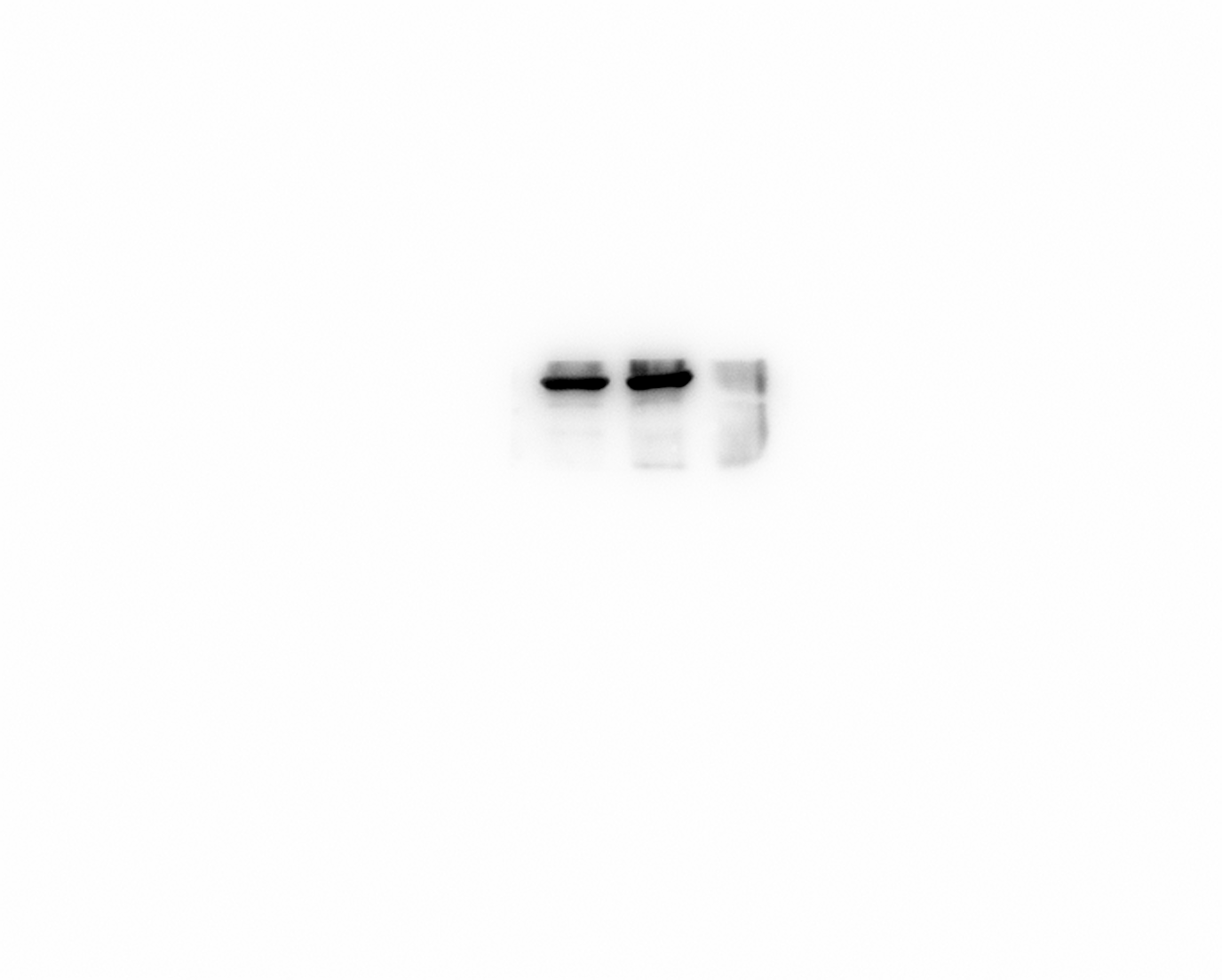


ALP staining

7 d

NC


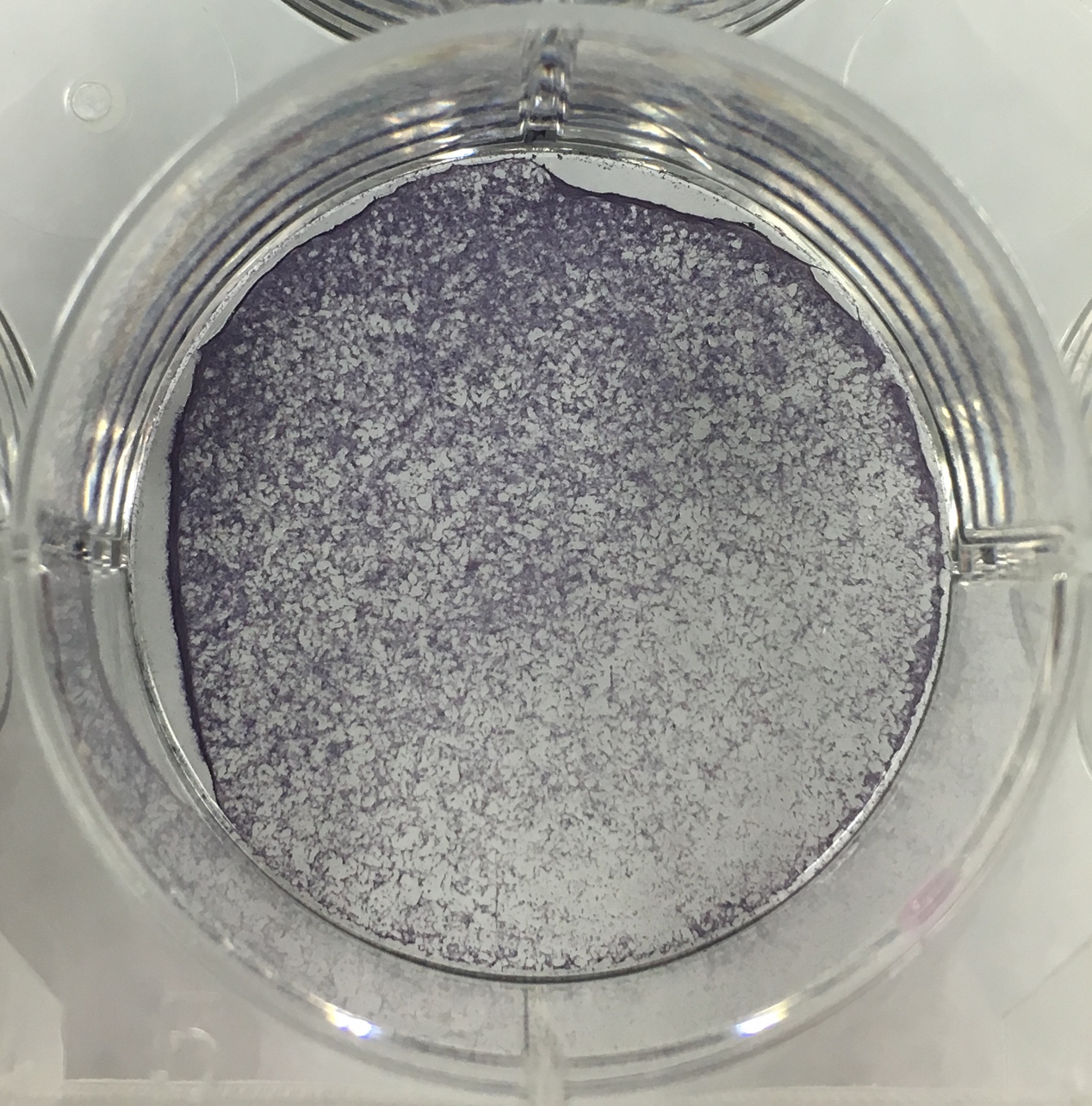


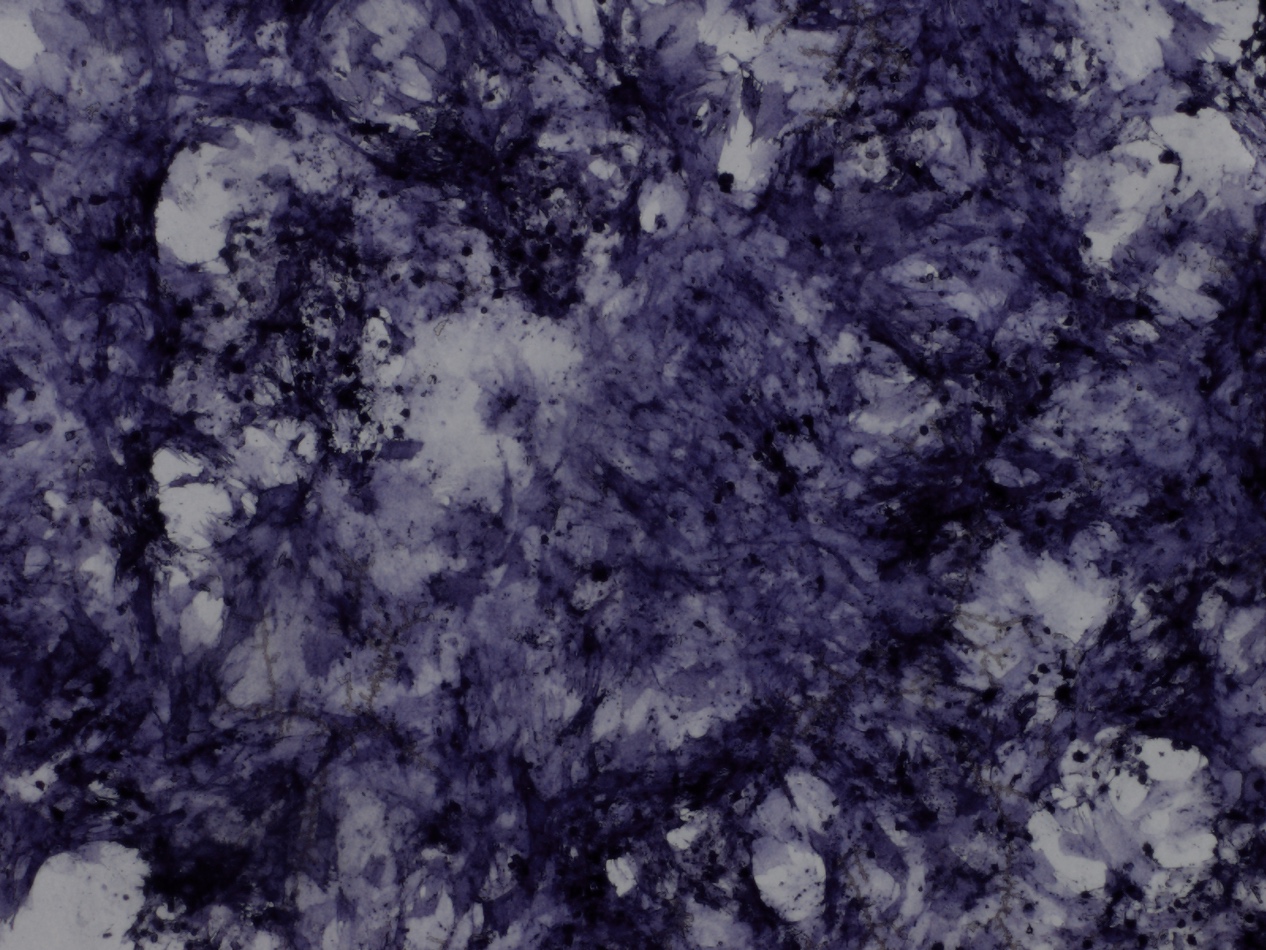


GARP-sh


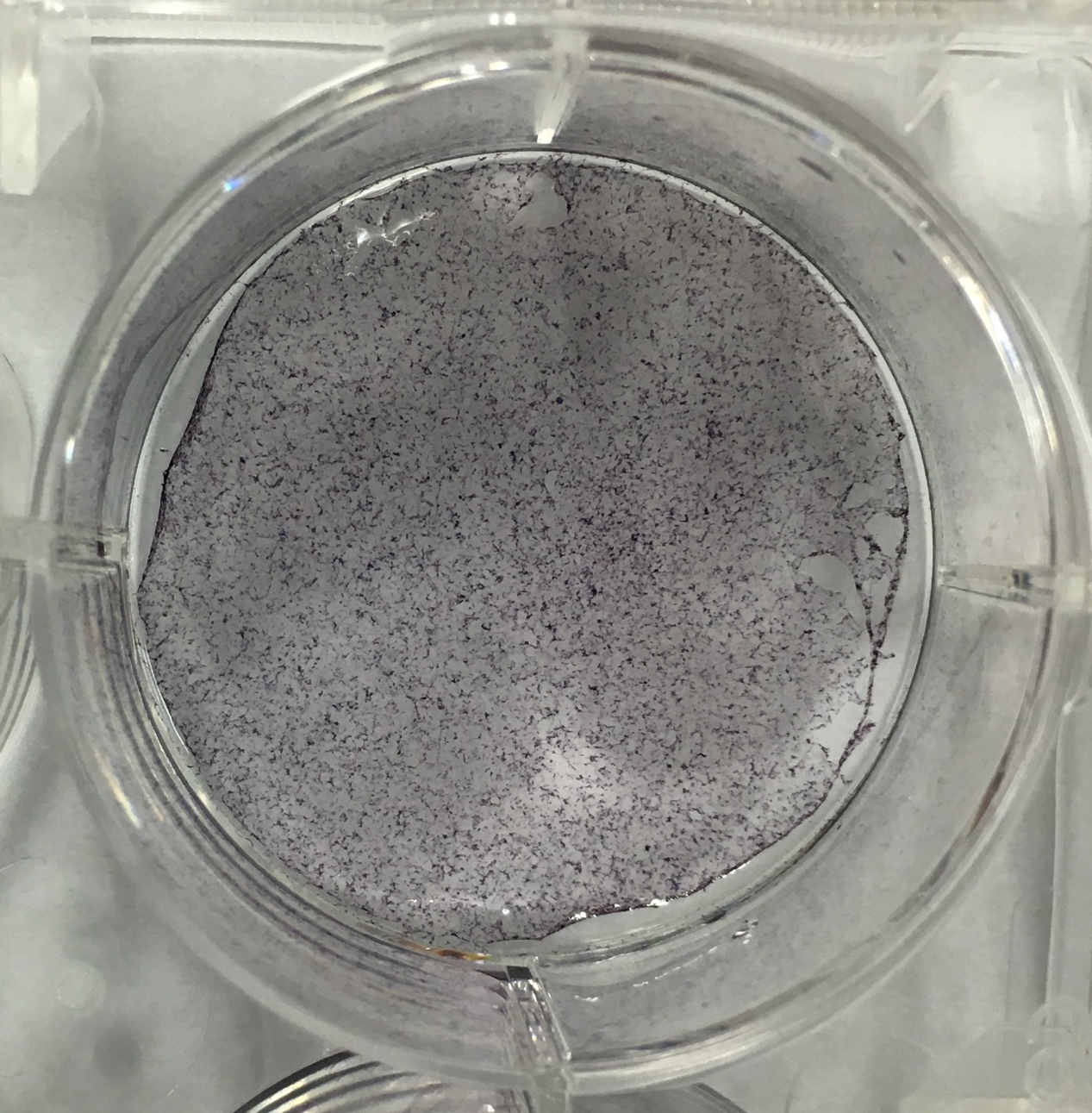


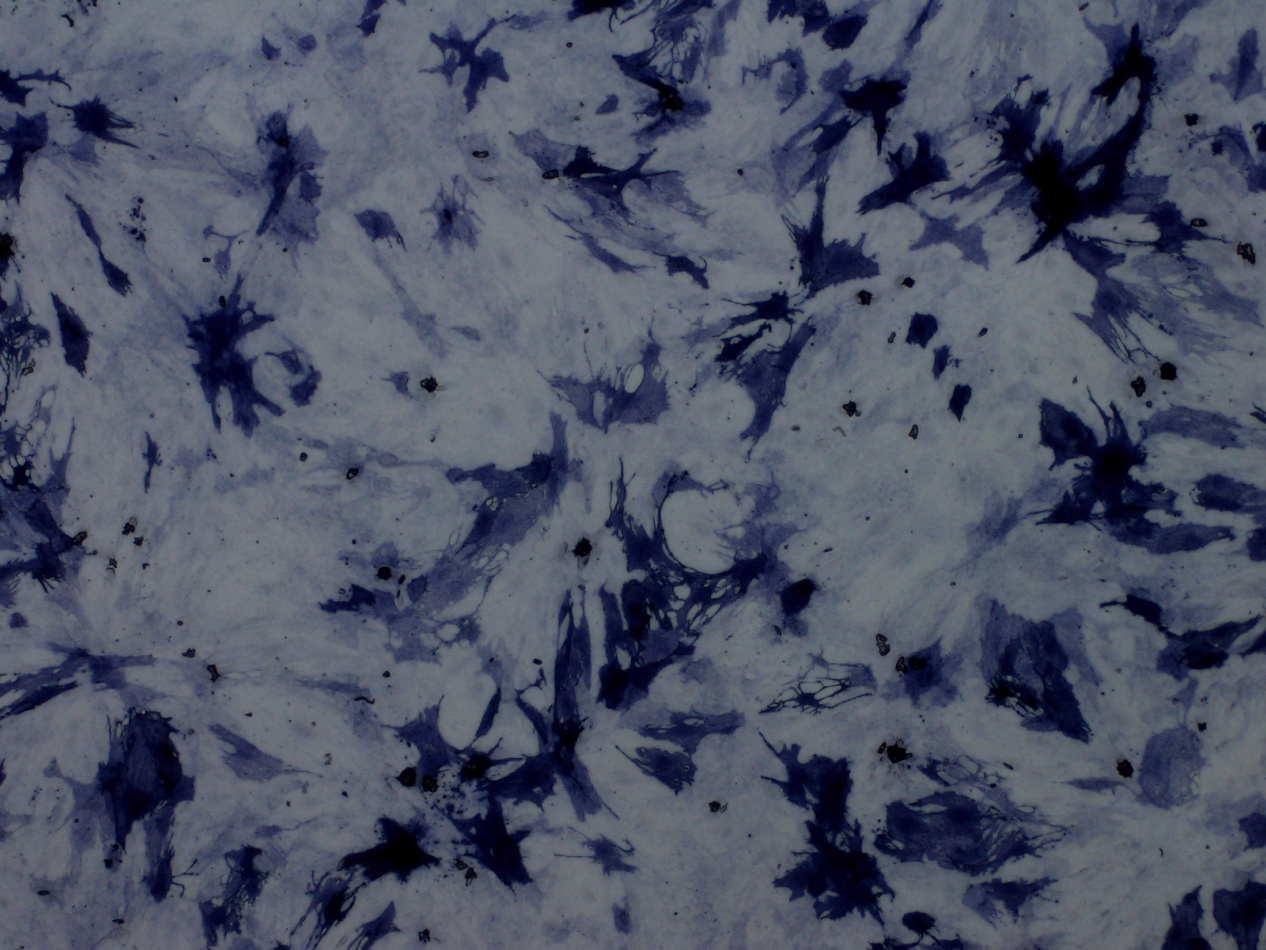


14 d

NC


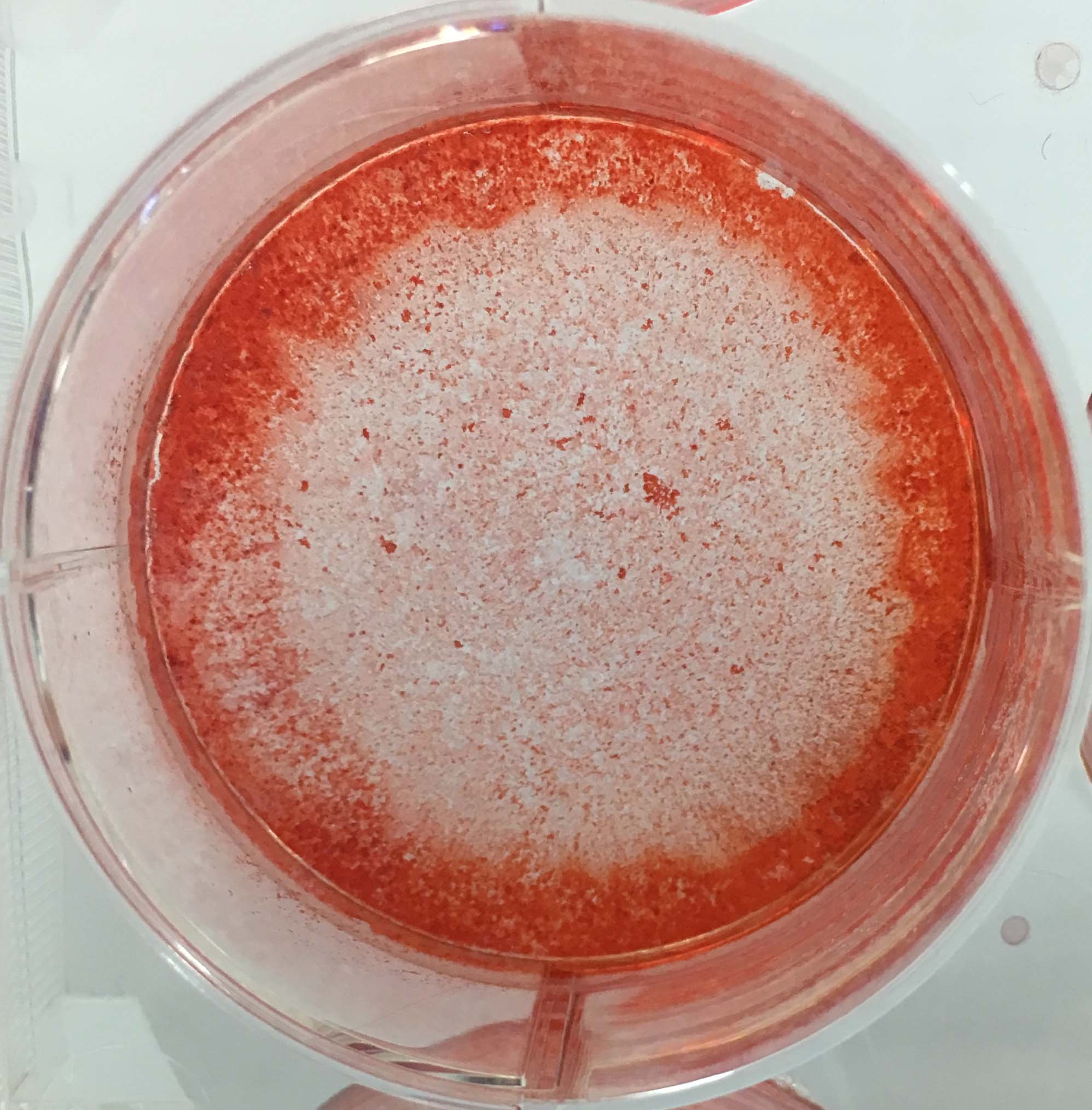


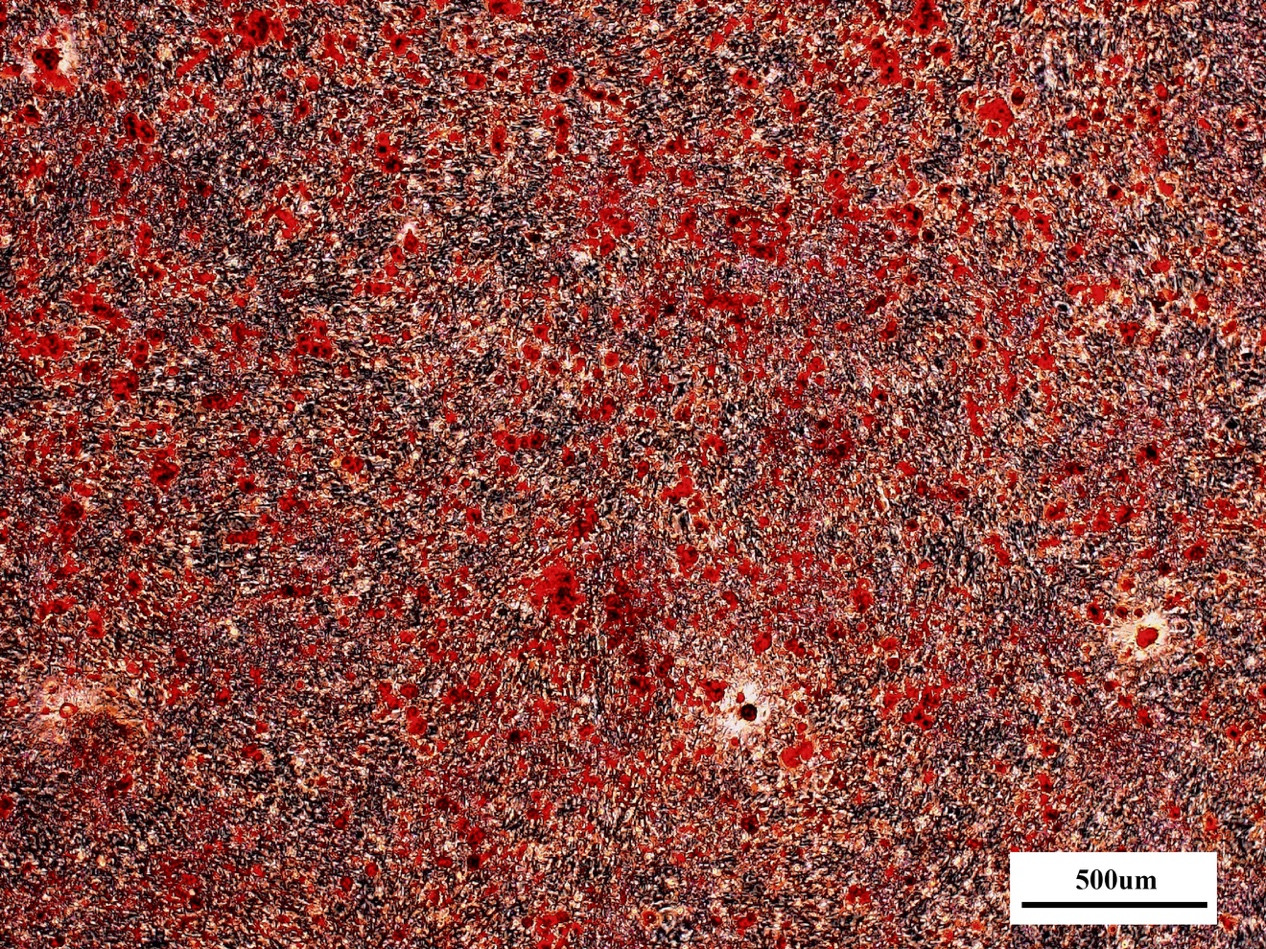


GARP-sh


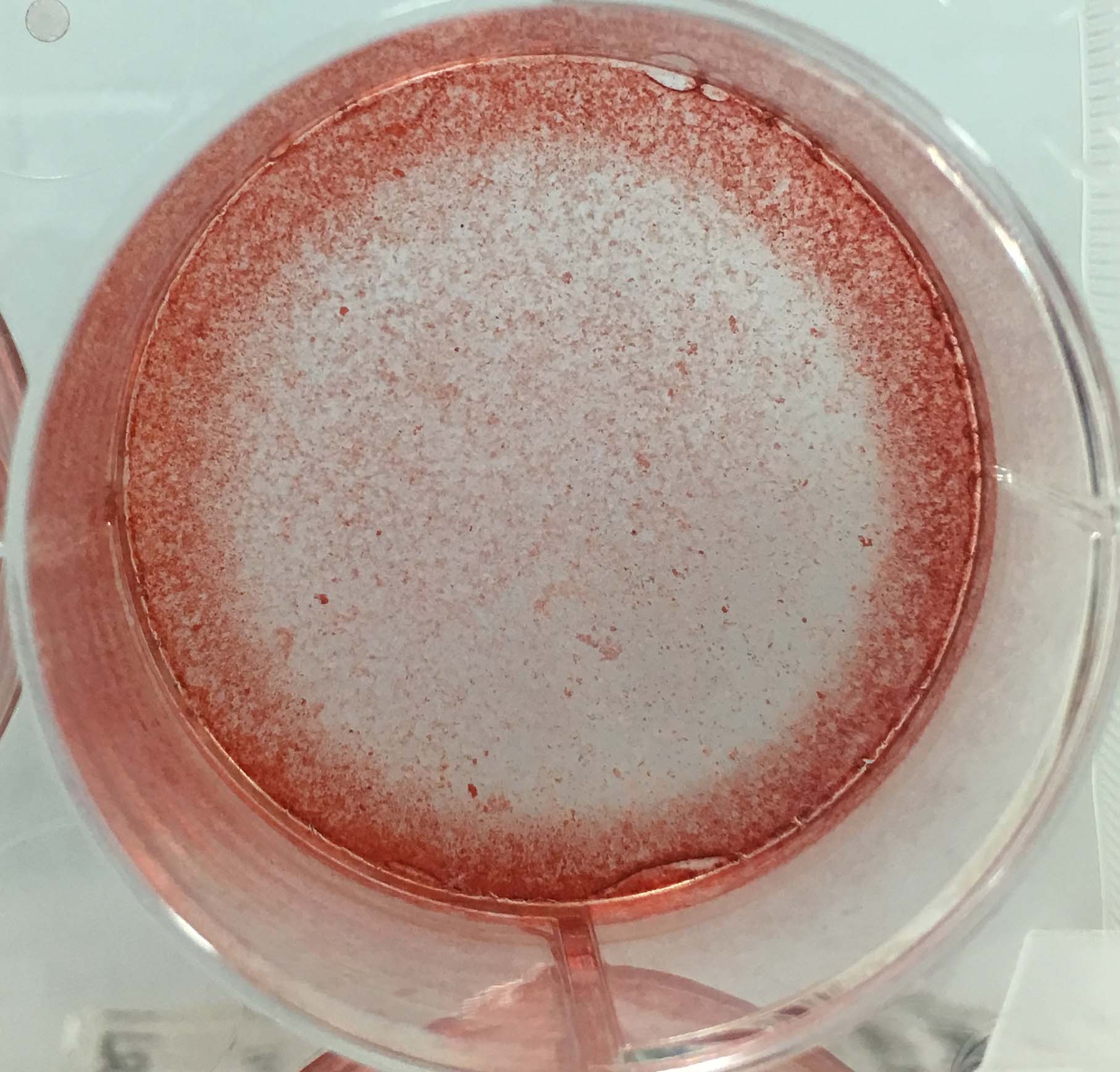


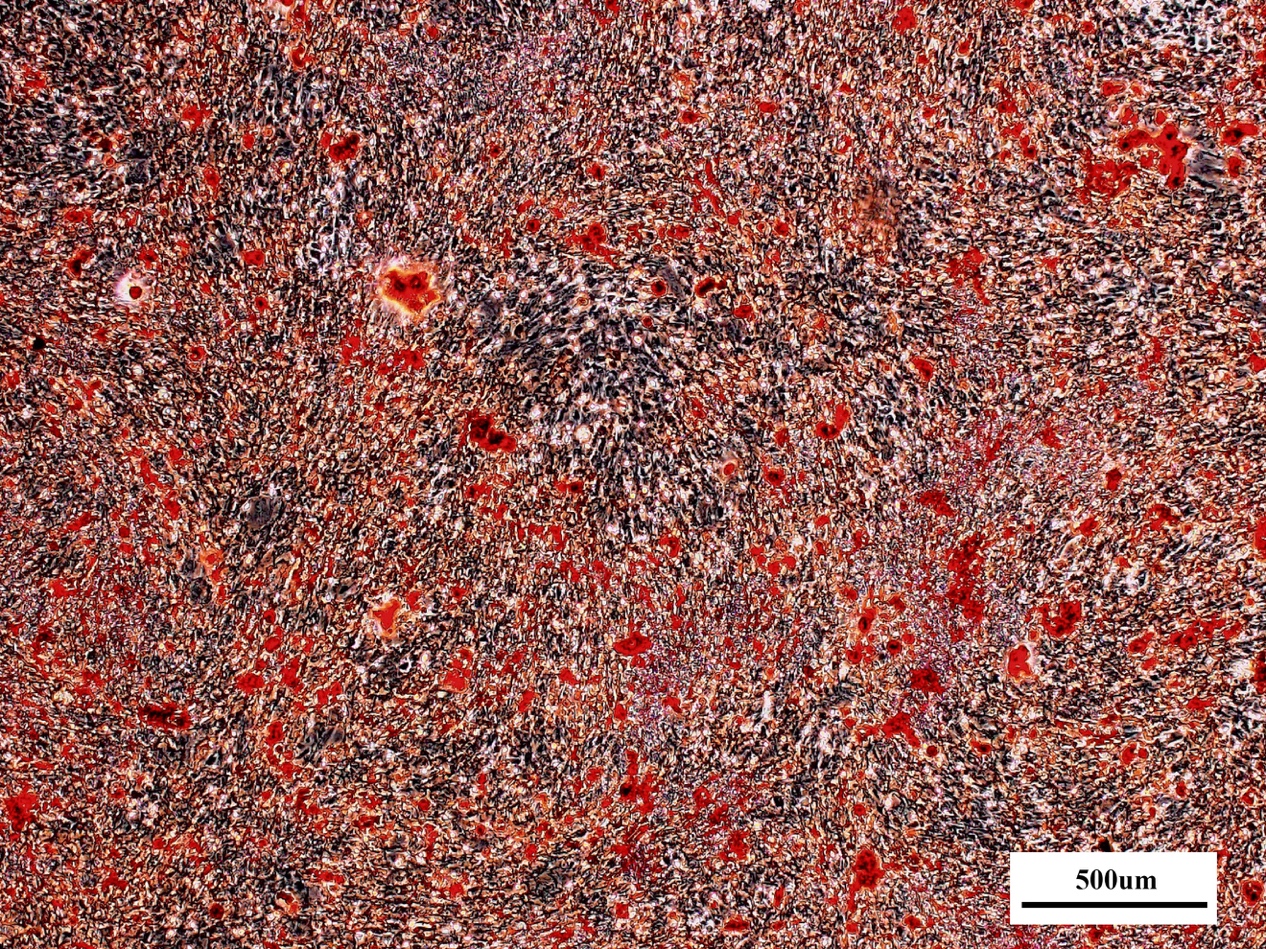

Supplement: Dataset S3 [file peerj-07-6993-s003.docx]
